# Implication of *S*-d-Lactoylglutathione in the Spontaneous Cysteine *S*-Glutathionylation and Lysine *N*-Lactoylation of *Arabidopsis thaliana* NAD-Dependent Glyceraldehyde-3-Phosphate Dehydrogenase

**DOI:** 10.3390/ijms26199673

**Published:** 2025-10-03

**Authors:** Camille Clément, Sonia Dorion, Natalia V. Bykova, Vincent Fetterley, Elvis Branchini, Charlie Boutin, Laurent Cappadocia, Jean Rivoal

**Affiliations:** 1 Institut de Recherche en Biologie Végétale, Université de Montréal, Montréal, QC H1X 2B2, Canada; camille.clement@umontreal.ca (C.C.); sonia.dorion@umontreal.ca (S.D.); vincent.fetterley@hotmail.com (V.F.); elvis.branchini@umontreal.ca (E.B.); charlie.boutin@umontreal.ca (C.B.); 2 Morden Research and Development Centre, Agriculture and Agri-Food Canada, Morden, MB R6M 1Y5, Canada; natalia.bykova@agr.gc.ca; 3 Département de Chimie, Université du Québec à Montréal, Montréal, QC H2X 2J6, Canada; cappadocia.laurent@uqam.ca

**Keywords:** *S*-d-lactoylglutathione, glyceraldehyde-3-phosphate dehydrogenase, post-translational modification, *S*-glutathionylation, *N*-lactoylation, cysteine, lysine

## Abstract

The glyoxalase pathway intermediate *S*-d-lactoylglutathione was recently implicated in protein post-translational modifications in animal systems. Here, we examined the spontaneous modification of the *Arabidopsis thaliana* cytosolic glyceraldehyde-3-phosphate dehydrogenase C1 (GAPC1) by this compound. Incubation of GAPC1 with *S*-d-lactoylglutathione resulted in the inhibition of enzyme activity. The inhibitory effect was concentration dependent and increased at alkaline pHs. Furthermore, the inhibition of GAPC1 by *S*-d-lactoylglutathione was favored by oxidative conditions and reversed by reduction with dithiothreitol. Analyses of the *S*-d-lactoylglutathione-treated protein by nanoLC-MS/MS revealed *S*-glutathionylation of its two Cys residues and *N*-lactoylation of six Lys residues. Protein structure predictions showed that the double *S*-glutathionylation is accommodated by the GAPC1 catalytic pocket, which likely explains enzyme inhibition. *N*-lactoylated sites overlap partially with previously reported *N*-acetylated sites at the surface of the GAPC1 tetramer. The efficiency of cytosolic glutaredoxin and thioredoxin isoforms was tested for reversing the *S*-d-lactoylglutathione-induced modification. In these assays, recovery of GAPC1 activity after inhibition by *S*-d-lactoylglutathione treatment was used as indicator of efficiency. The results show that both types of redoxins were able to reverse inhibition. We propose a model describing the mechanisms involved in the two types of post-translational modifications found on GAPC1 following exposure to *S*-d-lactoylglutathione. The possible involvement of these findings for the control over glycolytic metabolism is discussed.

## 1. Introduction

In their natural environment, plants are commonly exposed to a variety of stresses [1]. Because of their sessile nature, they have evolved biochemical mechanisms that allow them to efficiently detect harmful conditions and undergo changes in gene expression, physiology, or development to cope with stress [2,3,4]. A frequent consequence of plant exposure to stress is the production of reactive oxygen species (ROS) and methylglyoxal (MG) [5]. These compounds are usually generated at low levels and enzymatically detoxified under normal conditions [5]. ROS detoxification is achieved through non-enzymatic and enzymatic processes, in which the Foyer–Halliwell–Asada cycle plays a central part [6]. Under stress, ROS may have toxic effects, as well as signaling functions [7,8]. For example, stress-generated ROS can attack proteins and induce oxidation, which may lead to irreversible damage [9]. However, regulatory or signaling functions may result from oxidation of Cys leading to sulfenylated or *S*-glutathionylated residues [9]. The latter is a reversible post-translational protein modification (PTM) that occurs spontaneously through mechanisms such as the reaction of reduced glutathione (GSH) with a sulfenic acid Cys or a thiol-disulfide exchange between a protein thiol and oxidized glutathione (GSSG) [9,10,11]. *S*-glutathionylation has been implicated as a redox-sensitive regulator by activating or inhibiting a wide array of processes in diverse systems [12,13]. It also serves to protect Cys residues from irreversible oxidation during oxidative stress [12,13]. In plants, *S*-glutathionylation has been implicated in the inhibitory regulation of carbohydrate metabolism and signaling pathways [14,15,16,17,18,19].

MG is constitutively produced in plant cells, mainly as a by-product of triose phosphate metabolism. For instance, the triose phosphates in the glycolytic pathway and the photosynthetic Calvin–Benson–Bassham cycle can enzymatically or spontaneously lose a phosphate group, causing the formation of this harmful metabolite [5]. The cytotoxicity of MG is due to its highly reactive nature. It can generate advanced glycation end products by reacting with proteins, nucleic acids, or lipids [20]. The glyoxalase pathway is involved in its detoxification [5]. In this pathway, MG first undergoes a spontaneously condensation with GSH to generate hemithioacetal. This compound is then isomerized to *S*-d-lactoylglutathione (SLG) by glyoxalase I (GLO1, EC 4.4.1.5) [21,22]. Next, glyoxalase II (GLO2, EC 3.1.2.6) catalyzes the hydrolysis of SLG to D-lactate [23]. The latter is then used by a cytochrome-c-dependent D-lactate dehydrogenase (EC 1.1.2.4) to form pyruvate, which can be used in the citric acid cycle to produce energy and reducing power [24].

Lately, SLG has attracted interest due to evidence that it could act as a protein modifier. Studies conducted on mammalian cells substantiated the involvement of SLG in the non-enzymatic Lys *N*-lactoylation of proteins [25,26]. This PTM of the Lys ϵ-amino group with a D-lactate moiety is also called Lys lactylation [27,28]. The fermentative metabolite lactate can also promote lactoylation [28,29], leading to the modification of Lys by L-lactate [28]. In the case of SLG-induced modification, evidence for in vitro and in vivo protein modification was provided. In particular, incubation of purified recombinant histone H4 or phosphoglycerate kinase 1 with SLG for 24 h led to specific Lys *N*-lactoylation, as detected using MS/MS analysis [25]. Genome editing resulting in the loss of GLO2, thereby hindering methylglyoxal detoxification, raised cellular SLG as well as protein Lys *N*-lactoylation levels [25]. This same report showed an enrichment in the modification of glycolytic proteins, including glyceraldehyde-3-phosphate dehydrogenase (GAPDH, EC 1.2.1.12). These findings suggest that SLG-mediated Lys *N*-lactoylation could participate in metabolic feedback linking the activity of the glyoxalase pathway and glycolytic enzymes [25]. Specific mechanistic details for this feedback are beginning to emerge and may include inhibition of key enzymes [30]. In addition, there is evidence that SLG might be involved in *S*-glutathionylation. A study of human GLO2 showed that this enzyme promoted malate dehydrogenase (MDH, EC 1.1.1.37) and actin *S*-glutathionylation in the presence of SLG [31]. Interestingly, *S*-glutathionylation of these proteins also occurred, albeit at a lesser extent, in the presence of SLG alone [31]. It was proposed that human GLO2 could dock with MDH and actin and facilitate the modification of Cys residue(s) on these proteins. However, the spontaneous modification with SLG was not investigated and the modified residue(s) was(were) not identified.

Thus, there is evidence from the animal literature that SLG can induce spontaneous Lys *N*-lactoylation and Cys *S*-glutathionylation. The tendency of a protein Cys to be *S*-glutathionylated is influenced by its redox state, its p*K*a, and hence by the microenvironment in which the Cys is found [9]. For instance, alkaline pHs or the presence of basic amino acids in their microenvironment lower Cys p*K*as, promote deprotonation of the thiol group and favor *S*-glutathionylation reaction [9]. The removal of the glutathione moiety (deglutathionylation reaction) can occur in vitro in the presence of a strong reductant such as dithiotreitol (DTT). It can also be mediated by glutaredoxins (GRXs) and thioredoxins (TRXs), two related classes of disulfide reductases [9]. In these cases, the disulfide reductases are recycled to their reduced form in the presence of a regenerating system. For GRXs, this is achieved using GSH and NADPH-dependent glutathione reductase (GR, EC 1.8.1.7), whereas TRXs can be reduced in the presence of NADPH-dependent TRX reductase (NTR, EC 1.8.1.9).

GAPDHs are conserved glycolytic enzymes found in the plant cytosolic and stromal compartment of the plastids. Cytosolic NAD-dependent GAPDHs catalyze the phosphorylation and oxidation of glyceraldehyde-3-phosphate to generate 1,3-bisphosphoglycerate and NADH [32]. They are considered a major source of reducing power in the cytosol, and mutant studies have documented their implication in the control of respiratory rates, growth and reproductive development [33]. Additionally, various moonlighting activities have been assigned to GAPDHs (recently reviewed in [34]). The cytosolic GAPDH isoform 1 (GAPC1) of *Arabidopsis thaliana* (Arabidopsis) is known to be sensitive to various oxidative modifications, including *S*-glutathionylation [15,35,36]. GAPC1 only contains two Cys at positions 156 and 160 (also sometimes referred to as Cys^149^ and Cys^153^ [15,37] or Cys^155^ and Cys^159^ [35] in the literature). GAPC1 Cys^156^ is an acidic Cys exposed to the solvent and the active site of the enzyme. This residue is involved in reversible regulatory *S*-glutathionylation leading to the inhibition of enzyme activity [15]. In this research, we investigated the possibility that SLG could induce post-translational modifications of GAPC1.

## 2. Results

### 2.1. Inhibition of GAPC1 Activity by SLG and Factors Modulating This Effect

**Figure 1:**
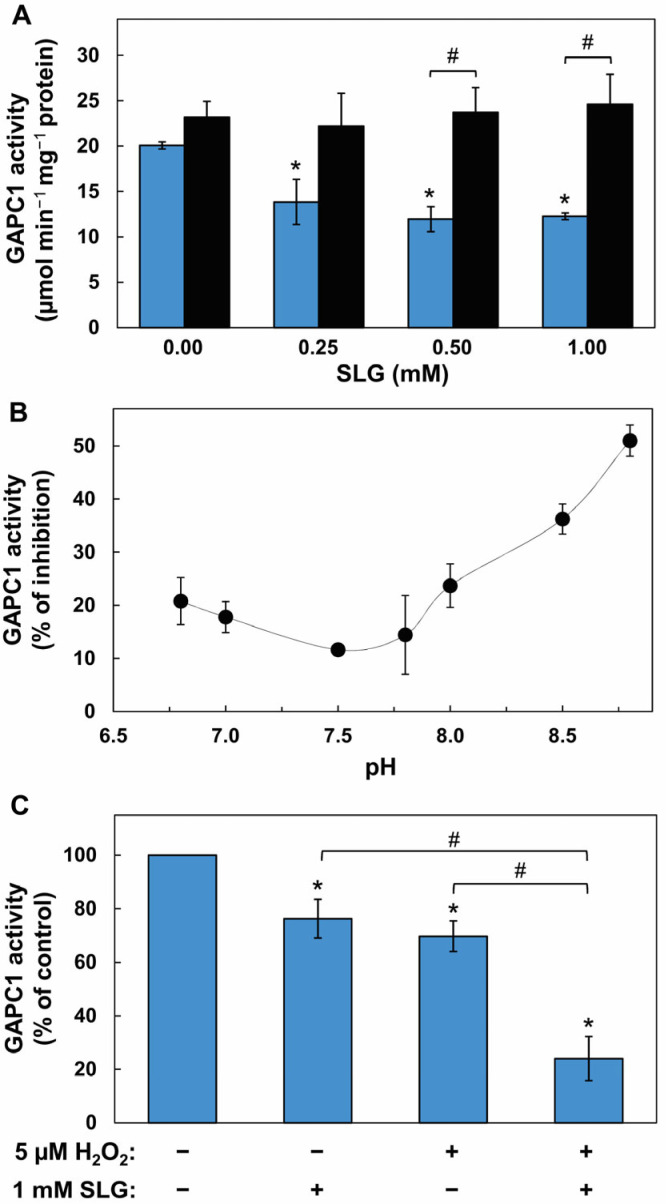
Characterization of parameters affecting the inhibition of GAPC1 activity by SLG. (**A**) Inhibition by SLG and its reversion by DTT. Recombinant GAPC1 was incubated for 10 min with concentrations of SLG between 0 and 1 mM. Remaining GAPC1 activity was measured immediately (blue bars), or after a further 10 min incubation with 10 mM DTT (black bars). (**B**) Effect of pH on inhibition by SLG. Recombinant GAPC1 was incubated for 10 min with or without 1 mM SLG in buffers of different pH values ranging between 6.8 and 8.8. GAPC1 activity was then measured on aliquots of these incubation mixtures. To calculate the % of GAPC1 inhibition, the activity without SLG was set at 100% for each pH value. (**C**) Effect of mild oxidative conditions on inhibition by SLG. Recombinant GAPC1 was first incubated for 20 min in the presence or absence of 5 µM H_2_O_2_. The protein was then treated for 10 min with or without 1 mM SLG. For the SLG control, H_2_O_2_ was replaced by water for 30 min. For the H_2_O_2_ control, GAPC1 was treated for 30 min with H_2_O_2_ and SLG was replaced by water for the 10 min incubation. An asterisk (*) marks a statistical difference between a treatment and the no SLG control (panel (**A**)) or a treatment and the no SLG, no H_2_O_2_ control (panel (**C**)) as determined using a Student’s *t*-test (*p* < 0.05). A hash sign (#) marks a statistical difference between two treatments identified by brackets as determined using a *t*-test (*p* < 0.05). Means ± SD of three replicates are shown.

We first investigated whether SLG could affect the activity of purified GAPC1 used as a target, and whether this effect could be sensitive to redox conditions. GAPC1 is sensitive to oxidative conditions, including *S*-glutathionylation at its catalytic site, which leads to a reversible inhibition of activity. An inactivation of enzyme activity can therefore be used as a proxy to study this PTM. The sequence encoding GAPC1 from Arabidopsis was cloned and the recombinant protein was expressed in *E. coli* (Figure S1). The protein was purified (Figure S2) and used to perform enzymatic assays. GAPC1 was incubated for 10 min with concentrations of SLG ranging from 0 to 1 mM (these conditions correspond to a SLG/GAPC1 molar ratio of up to 2000:1) and activity was measured on an aliquot of the mixture. Ten mM DTT was then added to the SLG-treated GAPC1. Following a further 10 min incubation, the resulting GAPC1 activity was measured. Incubation with SLG alone resulted in a significant reduction in GAPC1 activity at 1 mM SLG (Figure 1A–C).

The addition of 10 mM DTT to the SLG-inhibited GAPC1 reversed the inhibition at all SLG concentrations, leading to a significant recovery of activity at 0.5 and 1 mM (Figure 1A). Next, the influence of the medium pH over this effect of SLG was evaluated. For this, the pH of the SLG incubation mixture was adjusted between 6.8 and 8.8 (Figure 1B). For each pH value, we compared the activity of GAPC1 in the absence and in the presence of SLG using a SLG/GAPC1 molar ratio of 2000:1. The data show inhibition at all tested pHs. The effect of SLG was nevertheless most effective with pH values above 7.5. We then explored GAPC1 inhibition by SLG in the presence of H_2_O_2_. As demonstrated above (Figure 1A), the effect of SLG on GAPC1 is sensitive to DTT. GAPC1 is also known to be sensitive to oxidative conditions. We therefore investigated the effect of a mild oxidative treatment on GAPC1 inhibition by SLG. In a preliminary experiment, we exposed GAPC1 to 5 µM H_2_O_2_ (Figure S3). This led to a decrease in enzyme activity over time. We next evaluated whether this oxidative treatment could influence the inhibitory effect of SLG. Following a 20 min pretreatment with 5 µM H_2_O_2_ (or H_2_O as the control), GAPC1 was incubated with 1 mM SLG (or H_2_O as control) for a further 10 min. Enzyme activity was then measured (Figure 1C). GAPC1 was inhibited by H_2_O_2_ and by SLG in separate incubations. However, the data show that GAPC1 inhibition obtained with successive treatments with H_2_O_2_ and SLG was more important than those obtained with SLG or H_2_O_2_ alone. This indicates that the inhibition of GAPC1 activity by SLG increases under oxidative conditions.

### 2.2. Inhibition of GAPC1 by SLG Is Associated with Covalent Modifications

To further characterize the inhibitory effect of SLG on GAPC1, we investigated whether this compound could be involved in PTM(s). *S*-glutathionylation and *N*-lactoylation can be characterized with the help of nanoLC-MS/MS analyses, enabling the identification of modified amino acid residues. We therefore prepared samples by incubating GAPC1 with SLG. After trypsin digestion, tryptic peptides were subjected to nanoLC-MS/MS analysis.

#### 2.2.1. SLG Induces GAPC1 S-Glutathionylation

**Figure 2:**
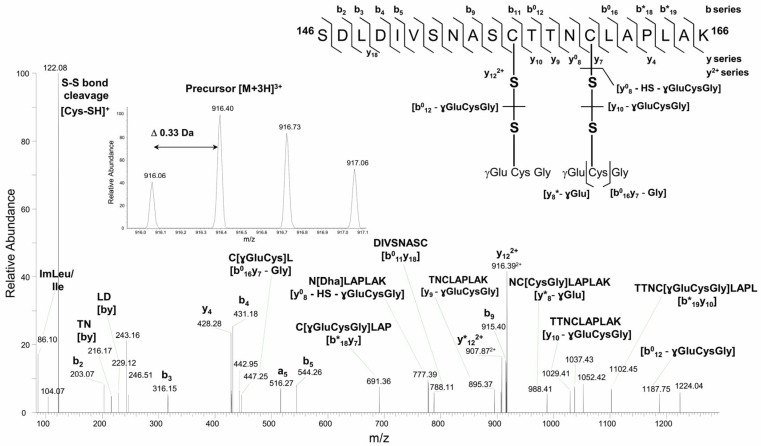
Higher-energy collision dissociation (HCD) tandem mass spectrum of the precursor peptide ion [M + 3H]^3+^ at *m*/*z* 916.06^3+^ reveals the presence of two *S*-glutathionylation sites at Cys^156^ and Cys^160^ residues. Sequence specific b- and y-type fragment ion signals, and a-type ions that derive from the corresponding b-type ion by losing CO, identify the peptide sequence ^146^SDLDIVSNASCTTNCLAPLAK^166^ (**top right corner**). The peaks denoted as b^0^ and y^0^, or b* and y* are the result of water (−18 Da) or ammonia (−17 Da) loss from corresponding ions, respectively. Internal peptide fragments labeled with “by” nomenclature indicate ions formed by double cleavage events in the peptide backbone, specifically cleavages on both the N-terminal (b) and C-terminal (y) sides of the fragment [38]. The spectral portion of precursor ion MS scan with isotopically resolved peaks indicating the parent ion charge state [M + 3H]^3+^ is shown in the insert.

Both GAPC1 Cys (at positions 156 and 160) are carried by the same tryptic peptide (^146^SDLDIVSNAS**C**TTN**C**LAPLAK^166^). The analysis of non-reduced samples containing GAPC1 treated with SLG revealed *S*-glutathionylation modifications of the two Cys residues (Figure 2).

For instance, in Figure 2, the fragment ion y_12_^2+^ at *m*/*z* 916.39^2+^ contains both glutathione modifications of the main peptide intact, whereas the fragment ion y*_8_ at *m*/*z* 988.41^+^ resulted from the partial fragmentation of glutathione at Cys^160^ residue with a neutral loss of 129 Da (ɣGlu). Specific fragmentation features of *S*-glutathionylation sites included the conversion of *S*-glutathionylated Cys^160^ into a dehydroalanine (Dha) moiety due to the loss of both glutathione (−305.07 Da) and –HS group from Cys residue (−33 Da) via the β-elimination mechanism. In [b^0^_12_ − glutathione] cysteinyl is a radical, whereas, in [y_10_ − ɣGluCysGly], the loss of glutathione during fragmentation involves a protonated form (−305.07 Da).

#### 2.2.2. SLG Also Induces Lys N-Lactoylation on GAPC1

**Figure 3:**
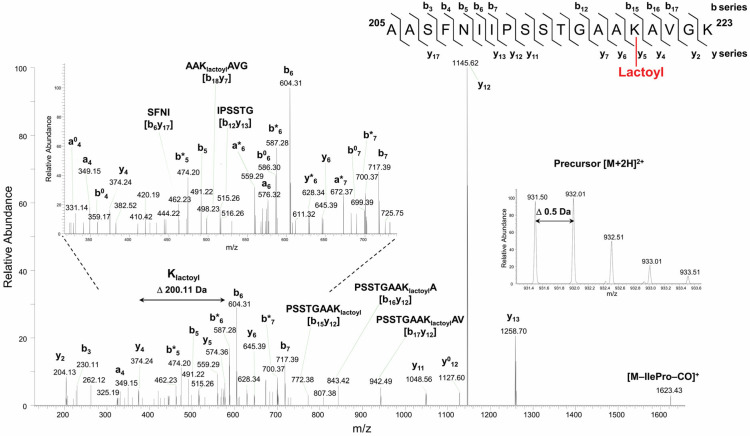
HCD fragmentation spectrum of the precursor peptide ion [M + 2H]^2+^ at *m*/*z* 931.50^2+^ obtained during nanoLC-MS/MS analysis identifies an *N*-lactoyl Lys modification site at Lys^219^. Sequence specific b- and y-type fragment ion signals, and a-type ions that derive from the corresponding b-type ion by losing CO, identify the peptide sequence ^205^AASFNIIPSSTGAAKAVGK^223^ (**top right corner**). The lactoyl Lys modification site was assigned by the 200.11 Da mass difference between y_4_ and y_5_ ions of C-terminal peptide fragments. The peaks denoted b^0^ and y^0^, or b* and y* are the result of water (−18 Da) or ammonia (−17 Da) loss from corresponding ions, respectively. Internal peptide fragments labeled with “by” nomenclature indicate ions formed by double cleavage events in the peptide backbone, specifically cleavages on both the N-terminal (b) and C-terminal (y) sides of the fragment [38]. The expanded region of the product ion spectrum showing detailed fragmentation pathways of *N*-lactoylated peptide is given on the (**top left**) side of the panel. The spectral portion of precursor ion MS scan with isotopically resolved peaks indicating the parent ion charge state [M + 2H]^2+^ is shown in the insert.

GAPC1 incubated with SLG was also analyzed in an attempt to find evidence for the possible modification of Lys residues. We were able to detect the *N*-lactoylation of a total of 6 Lys residues. An example of a fragmentation spectrum obtained with tryptic peptide ^205^AASFNIIPSSTGAAKAVGK^223^ carrying *N*-lactoylated Lys^219^ is shown in Figure 3. Evidence for other *N*-lactoylated residues (Lys^76^, Lys^190^, Lys^198^, Lys^255^ and Lys^310^) is shown in Supplementary Figures S4–S8.

In the fragmentation spectrum shown in Figure 3, internal fragment ions b_18_y_7_, b_15_y_12_, b_16_y_12_, and b_17_y_12_, as well as the sequence specific y_5_ to y_13_ and b_15_ to b_17_ ions, provided high-confidence evidence for the assignment of lactoyl Lys modification site at Lys^219^ residue. A Pro effect on the peptide fragmentation pattern was clearly observed and was enhanced by the presence of Ile residue. This selective fragmentation leading to the preferential cleavage of the peptide bond on the N-terminal side of Pro residue generated abundant y_12_-ion-containing modified *N*-lactoyl Lys residue. Moreover, a peptide scrambling effect [39] resulted in sequence rearrangement followed by the loss of the Ile-Pro pair and -CO (28 Da). A singly protonated y-type ion was detected at *m*/*z* 1623.43^+^, which corresponded to the rearranged sequence SSTGAAK_lactoyl_AVGKAASFNI containing the intact *N*-Lactoyl Lys^219^ modification.

### 2.3. Modeling of the SLG-Induced S-Glutathionylation Pattern on the GAPC1 Structure

**Figure 4:**
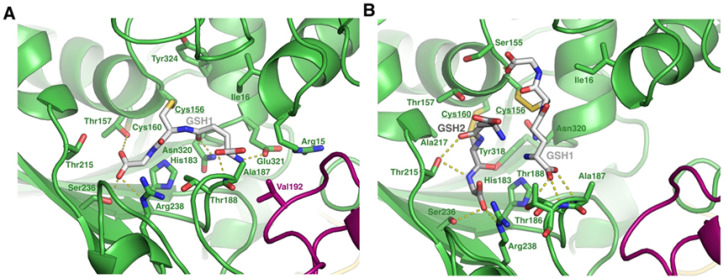
AlphaFold3 predicted structure of tetrameric GAPC1 *S*-glutathionylated on either (**A**) Cys^156^ alone or (**B**) both Cys^156^ and Cys^160^. GSH1 is colored in white and corresponds to the glutathione covalently attached to Cys^156^, whereas GSH2 is colored in grey and corresponds to the glutathione covalently attached to Cys^160^. Modified residues appear underlined in the figure. The purple and green colors represent two different subunits. Residues near GSH1 or GSH2 are in stick representation. H bond and salt bridge interactions are represented as yellow dashed lines.

Although not unique in the literature [40,41] the double *S*-glutathionylation pattern observed in Figure 2 is uncommon. We therefore investigated how the modification of both GAPC1 Cys residues could be accommodated by the protein. For this, the structure of the GAPC1 tetramer was predicted using Alphafold3 (v3.0.0). Since the single modification of Cys^156^ is the most commonly observed GAPC1 *S*-glutathionylation, we first analyzed the AlphaFold3-predicted structure of tetrameric GAPC1 modified on Cys^156^ (Figure 4A).

As expected, the GSH moiety on Cys^156^ is well accommodated by the structure. The model reveals that it sits in a cavity where it establishes a salt bridge with Arg^238^, H bond interactions with the side chains of Thr^157^, Ser^236^, Glu^231^ and Asn^320^, and an H bond with the main chain Ala^187^. Attempts to model GAPC1 solely modified on Cys^160^, which is localized in a hydrophobic environment, resulted in a mixture of accommodated and non-accommodated GSH. In the latter case, clashes were observed between GAPC1 residues and GSH. However, in the AlphaFold3-predicted structure of tetrameric GAPC1 doubly glutathionylated on Cys^156^ and Cys^160^ (Figure 4B), the two GSH molecules are accommodated by the structure and are located close to one another. Accommodation of both GSHs is permitted by a slight movement of the N-terminal helix where both Cys^156^ and Cys^160^ are located. In this conformation, both molecules interact with adjacent residues. Specifically: GSH1—attached to Cys^156^—interacts with the main chains of Cys^156^, Thr^188^ and Ala^187^ as well as the side chain of Thr^188^, whereas GSH2—attached to Cys^160^—interacts with the side chains of Thr^215^, Ser^236^ and Arg^238^.

### 2.4. Modeling of N-Lactoylated GAPC1 and Sequence Features Around N-Lactoylated Lys Residues

The *N*-lactoylated Lys residues were localized on the tetrameric structure of GAPC1 using Alphafold3. Analysis of the predicted structure (Supplementary Figure S9) revealed that Lys^76,190,198,219,255 or 310^ are relatively dispersed on the protein surface. In all cases, the primary amine of the side chain is oriented towards the solvent, supporting the idea that those residues are accessible prior to their lactoylation.

**Figure 5:**
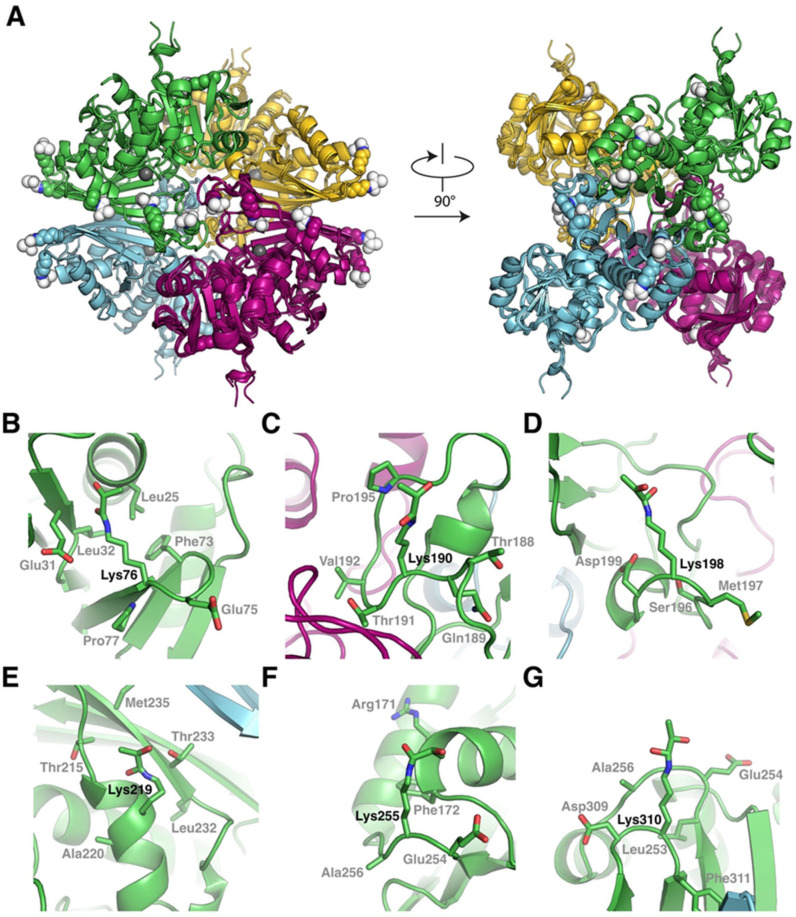
Structural impact of Lys *N*-lactoylation on GAPC1. (**A**) Structural alignment of AlphaFold3 predicted structures of non-modified tetrameric GAPC1 and GAPC1 individually lactoylated on Lys^76, 190, 198, 219, 255 and 310^. The catalytic Cys of GAPC1 is indicated with a grey sphere in each subunit. *N*-lactoylated Lys residues are represented as spheres and colored based on their originating subunit. In all cases, the lactoyl moieties are in spherical representations colored in white. (**B**–**G**) Close-ups of the structural context of GAPC1 individually lactoylated on (**B**) Lys^76^, (**C**) Lys^190^, (**D**) Lys^198^, (**E**) Lys^219^, (**F**) Lys^255^ and (**G**) Lys^310^.

To understand the structural context of the lactoylated Lys residues, we predicted the GAPC1 tetramer structure individually lactoylated on individual Lys^76,190,198,219,255 or 310^. In all cases, GAPC1 adopts the same tetrameric arrangement (Figure 5A). Furthermore, all structures are superimposable to the wild-type structure with the root mean square deviation always inferior to 0.5 Å, suggesting that *N*-lactoylation of individual Lys residues does not significantly perturb the overall or local structure of GAPC1. In particular, this implies that modified residues Lys^190,198 and 219^, which are localized within 25 Å of the GAPC1 active site, have no major impact on its structure. Further analyses of the structures reveal no strong similarity in the structural context of the *N*-lactoylated Lys residues (Figure 5B–G). Nevertheless, we note the presence of a Glu residue in the position preceding the Lys and the presence of a Phe close to the aliphatic portion of the Lys for Lys^76 and 255^ (compare Figure 5B,F).

We wanted to further evaluate whether SLG-associated modifications of GAPC1 Lys residues were associated with a local primary sequence pattern. For this, the sequences ranging from positions −10 to +10 around the modified Lys residues identified above were analyzed using the WebLogo3 tool (v3.7) [42]. Lys modifications were associated with an aliphatic residue at position +2 and a tendency (4/6) towards Val at this position (Supplementary Figure S10A). For comparison, a similar analysis was conducted on Lys residues for which there was no evidence of *N*-lactoylation in the GAPC1 sequence (Supplementary Figure S10B). The data show that the sequence around non-*N*-lactoylated Lys residues appears featureless.

### 2.5. SLG Inhibition of GAPC1 Is Reversible In Vitro by Treatments with Redoxins

**Figure 6:**
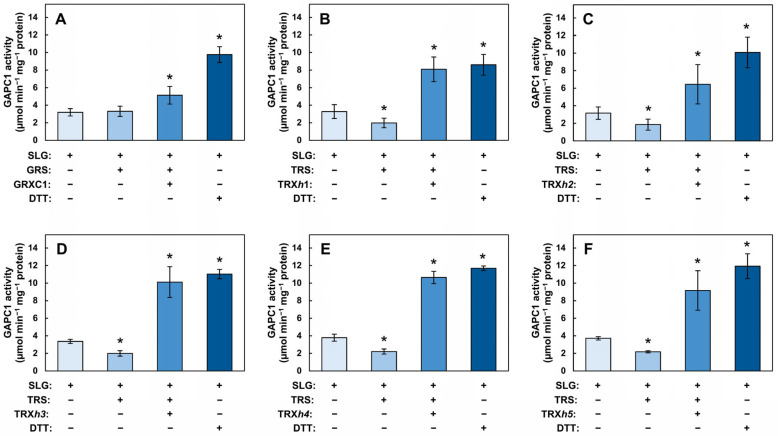
The inhibition of GAPC1 by SLG can be reversed by GRXC1 and TRXs *h*1–*h*5. Reactivation was achieved with (**A**) GRXC1, (**B**) TRX*h*1, (**C**) TRX*h*2, (**D**)TRX*h*3, (**E**) TRX*h*4 and (**F**) TRX*h*5. In all the panels, recombinant GAPC1 was first treated with SLG. In panel (**A**), GAPC1 was then further incubated for an additional 40 min with or without GRS (containing commercial GR, GSH and NADPH), or with GRS + 15 µM GRXC1, or with 10 mM DTT. In panels (**B**–**F**), GAPC1 was further incubated for an additional 30 min with or without TRS (containing NTRA and NADPH), or with TRS + 20 µM TRX or with 10 mM DTT. Following these incubations, GAPC1 activity was measured on an aliquot of the incubation medium. In all the panels, + and − signs below each bar indicate incubation conditions. Within each panel, different colors indicate different treatments and an asterisk (*) marks a statistical difference between a treatment and the SLG-inhibited enzyme, as determined using a Student’s *t*-test (*p* < 0.05).

The above experiments provided evidence that the inhibitory effect of SLG on GAPC1 activity was related to *S*-glutathionylation and could be reversed by DTT. GRXs and TRXs have the ability to remove GSH moieties from proteins in vitro and are likely involved in the physiological reduction of mixed disulfide bridges. However, so far, observations for the involvement of GRXs and TRXs in this reaction have been made using GAPC1 solely modified on the catalytic Cys [15,37]. The ability of the redoxins to deglutathionylate may be negatively affected because of the occurrence of two adjacent *S*-glutathionylated residues or by restricted access to the more buried vicinal Cys. In addition, the interference of nearby *N*-lactoylated residues may also impact enzymatic deglutathionylation. More generally, this issue is related to the possibility of reversing SLG-induced *S*-glutathionylation using physiologically relevant cytosolic redoxins. We therefore investigated the reversibility of GAPC1 inhibition by SLG using a glutaredoxin (GRXC1) and five TRXs (TRX*h1*–*h5*) (Figure 6). In these assays, we used a GRX recycling system (GRS) and TRX recycling system (TRS), respectively, corresponding to NADPH-dependent recycling systems for GRXC1 and TRXs. The GRS contained commercial GR, NADPH and GSH whereas the TRS contained recombinant NADPH-dependent thioredoxin reductase A (NTRA) and NADPH. The sequences of all the recombinant proteins used in these assays are shown in Supplementary Figure S1 and their purification is shown in Supplementary Figures S11 (GRXC1), S12 (NTRA) and S13 (TRXh1–h5). Control incubations were carried out in the presence of the redoxin but in the absence of their recycling system to verify the dependence of the reactivation on GRS or TRS. An additional control for reactivation was carried out by incubating SLG-treated GAPC1 with DTT, shown above to reverse SLG-induced inhibition.

In these experiments, the reactivation of SLG-inhibited GAPC1 was compared to the activity of the inhibited enzyme. Arabidopsis recombinant GRXC1 was first used to reactivate SLG-treated GAPC1 (Figure 6A). Incubation with GRS alone did not lead to any reactivation, whereas the addition of GRXC1 plus GRS significantly increased GAPC1 activity. A higher level of reactivation was obtained with DTT. GRXC1 was therefore able to reverse inhibition in a GRS-dependent manner. Under the conditions used, GRXC1 reactivation is, however, not as effective as DTT. The reactivation of the SLG-treated GAPC1 was also tested with five cytosolic TRXs. Recombinant Arabidopsis TRX*h*1 (Figure 6B), TRX*h*2 (Figure 6C), TRX*h*3 (Figure 6D), TRX*h*4 (Figure 6E) and TRX*h*5 (Figure 6F) were used with or without the TRS. In these experiments, incubation with the TRS was accompanied with a small but significant decrease in activity. However, when a TRX was added to the TRS, a strong reactivation of GAPC1 was achieved. Taken as a whole, these results indicate that GRXC1 and the five TRXs tested were able to reactivate SLG-inhibited GAPC1, and that, under the experimental conditions used, this reactivation was dependent on the TRS.

## 3. Discussion

SLG is a key intermediate in the glyoxalase pathway, which is involved in MG detoxification in biological systems. SLG is produced from hemithioacetal by GLO1 [21] and catabolized to D-lactate and GSH by GLO2 [23]. In recent years, the possibility that SLG could act as a protein modifier has emerged. Some evidence for this comes from studies of Human Embryonic Kidney 293 (HEK293) cells in which the deletion of GLO2 is accompanied by a rise in SLG and a large increase in Lys *N*-lactoylation when cells are treated with MG [25]. Moreover, a spontaneous in vitro *N*-lactoylation of Lys in the presence of SLG has been shown to occur on recombinant histone H4 and phosphoglycerate kinase [25]. Literature data also suggest that SLG could induce spontaneous *S*-glutathionylation [31]. Here we sought to further characterize the involvement of SLG as a protein modifier by investigating its role in the modulation of GAPC1 activity and structure. We found that an incubation of GAPC1 with SLG results in an inhibition of enzyme activity. This inhibition is pH dependent and sensitive to redox conditions. We observed a reversion of inhibition by DTT and an increase in SLG inhibition under oxidative conditions. Further examination of SLG-treated GAPC1 using nanoLC–MS/MS demonstrated covalent modifications of six Lys residues by *N*-lactoylation and two Cys residues by *S*-glutathionylation. Structural modeling studies revealed that the GAPC1 structure could accommodate the modification of two Cys and allowed the visualization of modified Lys at the protein surface. Below, we discuss our main findings and provide a hypothetical series of chemical reactions leading to the observed spontaneous modifications and inhibition of GAPC1. The relevance of our results for the regulation of GAPC1 functions is presented.

### 3.1. Inhibition of GAPC1 in the Presence of SLG Is Reversed by DTT

In vitro incubation of GAPC1 with SLG led to a decrease in enzyme activity, which was sensitive to redox conditions (Figure 1). The activity decrease due to SLG was reversed in a subsequent incubation with DTT (Figure 1A), whereas mild oxidative conditions brought about by 5 µM H_2_O_2_ enhanced inhibition by SLG (Figure 1C). Due to the reversibility of the inhibition by DTT, these results are compatible with the interpretation that SLG causes a redox modification of GAPC1, and they point to the possible involvement of *S*-glutathionylation. The latter takes place when a Cys residue forms a mixed disulfide bridge with reduced glutathione. GAPC1, like other proteins of the GAPDH family, is prone to Cys sulfenylation, an oxidized state that can be promoted by H_2_O_2_ and facilitate *S*-glutathionylation [15,35,43]. Moreover, we found that inhibition by SLG was increased under alkaline pH conditions (Figure 1B). Surface-accessible Cys residues in proteins can become deprotonated at physiological pH values (slightly above neutrality) [44]. It is the case, for example, with GAPC1 catalytic Cys^156^, which has a p*K*_a_ below pH 6 [15]. Thiol ionization makes Cys much more reactive and susceptible to oxidation and to *S*-glutathionylation [9,45]. Interestingly, GAPC1 vicinal Cys^160^ is more buried in the structure of GAPC1 and thus, is considered less available than Cys^156^ for PTMs [45]. This is consistent with observations in which incubation of oxidized GAPC1 with GSH leads to the modification by *S*-glutathionylation of its catalytic Cys and to enzyme inhibition [15,35]. Similar observations have been made with the chloroplastic A4 GAPDH isoform [46]. In related modification mechanisms, nitrosoglutathione and the bulkier oxidized form of GSH (GSSG) were shown to mediate the addition of a glutathione moiety on GAPCs [35]. So far, *S*-glutathionylation of GAPC1 has not been connected to any other protein modifier.

### 3.2. SLG Causes Spontaneous Lys N-Lactoylation and Cys S-Glutathionylation of GAPC1

**Figure 7:**
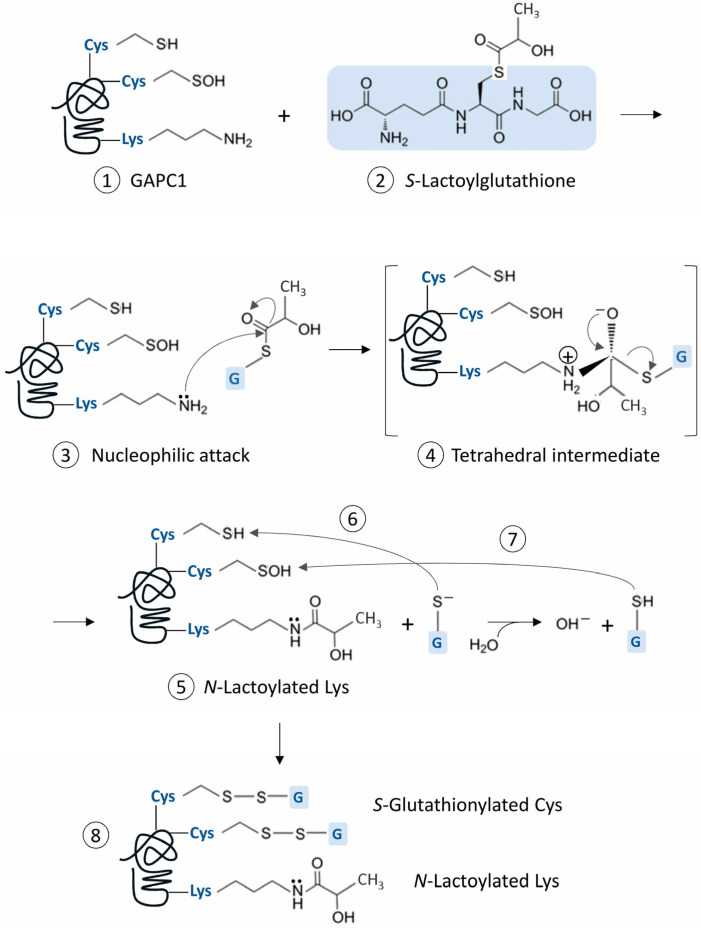
Scheme representing the hypothetical chemical reactions leading to the observed spontaneous Cys *S*-glutathionylation and Lys *N*-lactoylation of GAPC1. The representation of GAPC1 (**1**) carrying Cys residues in thiol and sulfenic acid forms is only shown to illustrate two possible pathways leading to *S*-glutathionylation and does not imply that they occur simultaneously. The C backbone of SLG’s glutathione moiety is highlighted in blue (**2**) and is subsequently symbolized by ‘G’ on a blue background in the rest of the figure. (**3**) The nucleophilic attack of a Lys ϵ-amino group on the carbon of SLG’s thioester bond leads to the formation of a tetrahedral intermediate (**4**), which is resolved by the *N*-lactoylation of the Lys residue (**5**) with a with a D-lactate moiety. This reaction releases glutathione thiolate (GS^−^), which can attack a Cys thiol (**6**) or become protonated in solution, generating GSH, which can modify a Cys sulfenic acid (**7**). Both reactions of GAPC1 with the glutathione moieties can lead to the *S*-glutathionylation of Cys residues (**8**).

The nanoLC-MS/MS analysis conducted after GAPC1 incubation with SLG revealed multiple modifications on the protein. Here, we report for the first time the Lys *N*-lactoylation of SLG-treated GAPC1 and the identification of six modified residues. The spontaneous *N*-lactoylation (an acylation reaction) and *S*-glutathionylation discussed below implies a series of reactions that logically first involves a S to N nucleophilic acyl substitution. The general chemical process causing this type of protein modification has been described [47] and a parallel can also be made with the mechanism involved in other Lys modifications [48]. Thus, we propose a possible series of reactions that can account for the two types of spontaneous PTMs that were found on GAPC1 (Figure 7).

In this scheme, the C atom engaged in the thioester bond of the SLG structure has electrophilic properties, since electrons are attracted to the neighboring oxygen. In a proper microenvironment, a protein Lys ϵ-amino group can then act as a nucleophile by attacking the C atom, leading to the formation of a tetrahedral reaction intermediate between SLG and the Lys side chain. This structure is resolved by a redistribution of electrons from the O atom leading to the acylation of the Lys side chain and the liberation of a the negatively charged GS^−^. The latter is highly reactive and can modify a Cys thiol, leading to *S*-glutathionylation [49]. It can also undergo protonation in solution and subsequently modify a Cys sulfenic acid. We found a greater inhibitory effect of SLG with increasing pH (Figure 1B). This observation could be influenced by the p*K*a value of the GSH thiol group (around 8.6 [50]), meaning that, in that pH range, 50% of the GSH liberated from the tetrahedral intermediate remains in its thiolate form. Interestingly, the generation of deprotonated GSH is part of the catalytic mechanism of glutathione-S-transferases (GSTs), [51]. This leads to a stabilization of GS^−^ at the enzyme active site and is seen as a preliminary step towards its activation and transfer to an electrophilic acceptor [51]. This mechanism is present in the Pi-class GSTs [52], which are known to be involved in protein *S*-glutathionylation in animals [53].

Compared to GSH and GSSG, SLG seems to generate a different pattern of *S*-glutathionylation. Indeed, our data also provide a rare example of GAPC1 *S*-glutathionylation on both its Cys (Figure 1A). The simplest explanation for this is that this pattern is due to the reaction of SLG with Lys residues generating GS^−^. The modification of both catalytic and vicinal Cys residues by glutathione, concomitant with the inactivation of enzyme activity and modification of protein conformation has however been detected in animal GAPDHs [40,41]. The methods used here do not allow for a quantitative evaluation of the proportion of modified GAPC1. Nevertheless, based on the well-known inhibition of the enzyme by *S*-glutathionylation, it appears likely that the modified enzyme would be inactive. Furthermore, *S*-glutathionylation of GAPC1 has also been shown to start the self-aggregation process of the protein, which may lead to irreversible insolubility [37].

GAPC1’s *N*-lactoylated Lys were mapped. They localize to exposed areas at the protein surface (Figure 5 and Figure S9). Some modified Lys localize near the two GAPC1 Cys residues. However, this does not seem to perturb the structure of this region. Nevertheless, this proximity could facilitate the diffusion of GS^−^ or GSH towards Cys residues. The six modified Lys on GAPC1 were associated with an aliphatic residue located two positions downstream in the sequence. Obviously, this feature will need to be confirmed in future studies of SLG-dependent *N*-lactoylation with other proteins. Nonetheless, it is worth noting that an aliphatic amino acid residue was also present at position +2 relative to the *N*-lactoylated Lys sites detected in human histone H4 (Ala) and phosphoglycerate kinase 1 (Leu) after in vitro treatment with SLG [25]. Another Lys modification, *N*-acetylation, has been described in rice GAPC1 [54] and *Capsicum annum* GAPCs [55] and was reported to inhibit Arabidopsis GAPC2, which shares 98% sequence identity with GAPC1 [56]. It is interesting to note the occurrence of a partial overlap between GAPC1’s *N*-lactoylated sites and *N*-acetylated sites in rice GAPC1 (Lys^76,190,219,255^ [54]), Arabidopsis GAPC2 (Lys^219,255^, [56]) and *C. annum* GAPCs (Lys^76,198^, [55]). At this time, the possible physiological relevance of SLG-induced *N*-lactoylation of GAPC1 is not known. At a physiological pH, Lys normally carries a positive charge. *N*-lactoylation or *N*-acetylation of the side chain would both suppress this charge. In turn, this change can potentially affect GAPC1’s activity, properties or functions. The fact that we were able to recover GAPC1 activity after reducing treatments (Figure 1A and Figure 6) suggests that *S*-glutathionylation is solely or principally responsible for the loss of activity following SLG treatment. However, our experiments do not allow us to completely rule out the participation of *N*-lactoylation in GAPC1 inhibition. Besides its role as a glycolytic pathway enzyme, GAPC1 has also been implicated in a number of moonlighting functions [57]. These include, among others, H_2_O_2_ signaling [58], the negative regulation of resistance to *Pseudomonas syringae* pathogenicity, cell death and autophagy [59], a function as DNA binding factor and/or transcriptional regulator of glycolytic genes [35,54], an activity as a transcriptional regulator of heat stress response [60] and involvement in RNA translation inhibition during the dark/light transition [61]. Several of these activities involve interactions of GAPC1 with partners and/or the modification of its subcellular localization [54,58,60]. In particular, *N*-acetylation of rice GAPC1 was shown to stimulate its translocation to the nucleus and its function as a transcriptional regulator [54]. As mentioned above, there is an overlap between *N*-acetylated and *N*-lactoylated sites on GAPDHs. This suggests a possible interaction between the two modifications. It will therefore be interesting to study whether there is a cross-talk between the two types of Lys modifications. In addition to *N*-acetylation and *N*-lactoylation, GAPC1 Lys^76^ has also been identified as the mono-ubiquitination site of E3 ubiquitin-ligase SEVEN IN ABSENTIA like 7 (SINAL7) [62]. SINAL7 is required for the nuclear translocation of GAPC1. Hence, if it occurs in vivo, *N*-lactoylation of Lys^76^ may also interact with GAPC1’s nuclear translocation. Further investigations will be required to understand the interplay between Lys^76^ PTMs.

### 3.3. SLG-Induced Inhibition of GAPC1 Can Be Reversed by GRXC1 and TRXs

Our results constitute the first report of an inhibition of GAPC1 activity by SLG. This effect was sensitive to DTT and H_2_O_2_ and was shown to be concomitant with an atypical *S*-glutathionylation pattern of the protein. GRXs and TRXs are known to catalyze protein deglutathionylation. GRXC1 and TRXs *h*1–*h*5 have been predicted to localize to the cytosol [63,64], while alternative localization may also take place for TRX*h*2 and *h*5 [65]. They are therefore likely physiological candidates for the reversion of GAPC1 *S*-glutathionylation. The TRXs and GRX used here display differences in their redox centers. The active site of TRXs *h*1 and *h*2 is WCGPC, while the WCPPC motif is found in TRXs *h*3–*h*5, and GRXC1 carries CGYC [65,66]. These proteins have a broad, overlapping substrate specificity that may be influenced by parameters such as electrostatic complementarity with their targets [67]. During their reaction, the redoxins become oxidized and they must be reduced to start a new catalytic cycle. Here, we showed that reactivation of SLG-inhibited GAPC1 could be achieved with GRXC1 and TRXs *h*1–*h*5 (Figure 6). In all cases, reactivation was dependent on the presence a redoxin recycling system. Thus, our in vitro assays provide evidence that multiple physiological redoxins, with different catalytic mechanisms, may be involved in the reversion of the atypical SLG-promoted GAPC1 *S*-glutathionylation pattern. This result is important since, in published trials of plant GAPDHs deglutathionylation, assays were carried out with enzymes carrying a single GSH moiety on the catalytic Cys [15,37,68]. Our results are consistent with the conclusion that the double *S*-glutathionylation and the *N*-lactoylation of GAPC1 do not prevent the redoxins’ capacity to reactivate GAPC1. They thus appear to be good candidates to conduct this reaction in vivo.

### 3.4. Physiological Relevance of SLG-Dependent PTMs of GAPC1

SLG is a key intermediate of the glyoxalase pathway, which is considered the main detoxification route for the highly reactive compound MG [69]. The latter is produced at low levels during normal operation of the glycolytic and photosynthetic pathways [5]. Multiple abiotic and biotic stress conditions have been shown to significantly increase MG production and the activity of the glyoxalase pathway [70,71,72,73,74]. Thus, MG detoxification under stress may raise cellular SLG levels. This situation alone is conducive to the *N*-lactoylation and *S*-glutathionylation of GAPC1, leading to its inhibition. In Arabidopsis, inhibition of GAPC1/2 activity has been linked to a redirection of carbohydrate catabolism towards the pentose phosphate pathway and NADPH generation, presumably as an adaptative mechanism to oxidative stress [75]. Multiple stresses can lead to a rise in cellular H_2_O_2_ [5]. As we showed (Figure 1), the combination of SLG and oxidative conditions increase the inhibition of GAPC1. Thus, we hypothesize that the generation of SLG by the glyoxalase pathway under stress could lead to the multiple PTMs of GAPC1 described in this report. In turn, these modifications could impact GAPC1 activity and/or affect its moonlighting functions. As discussed above, the partial overlap of *N*-lactoylated and *N*-acetylated sites on GAPC1 is intriguing. Given the implication of GAPC1 *N*-acetylation in the protein nuclear translocation, it will be interesting to examine the hypothesis that GAPC1 *N*-lactoylation modulates its migration to the nucleus and moonlighting functions.

## 4. Materials and Methods

### 4.1. Chemicals

Unless otherwise indicated, the chemicals, enzymes and buffers used in this study were of analytical grade and obtained from Fischer Scientific (Mississauga, ON, Canada) or Sigma-Aldrich (Oakville, ON, Canada). Sequences coding for (10×His)-tagged recombinant Arabidopsis *Thioredoxin h1* (*TRXh1*, *At3g51030*) and *NTRA* (*At2g17420*) were generated using DNA synthesis at Gene Universal (Newark, DE, USA). These constructs were made in the pET-19b vector and were transformed in competent *Escherichia coli* (BL21 DE3pLysS strain). The plasmids carrying the constructs were purified and verified via sequencing. The sequences of the recombinant proteins are shown in Figure S1. The construction of the plasmid expressing (6×His)-tagged recombinant Arabidopsis glutaredoxin C1 (GRXC1) used here was previously described [16].

### 4.2. Plasmid Constructions

Plasmids carrying Arabidopsis *GAPC1*, *thioredoxins h2* (*TRXh2*), *h3* (*TRXh3*), *h4* (*TRXh4*), and *h5* (*TRXh5*) were obtained from the Arabidopsis Biological Resource Center (ABRC) [76] (Table S1). The vector was pUNI51 for all cDNAs, except for *TRXh3*, which was in pENTR/SD/DTOPO. All recombinant DNA manipulations were carried out according to standard techniques [77]. PCR was used to amplify the coding sequences using primers listed in Supplementary Table S1. Amplicons were purified and digested with *Not1* (or *Xba1* for *TRXh3*). The digested inserts were ligated in frame with an N-terminal (6×His) tag into the pProExHTb vector previously digested with *EheI*/*NotI* (or with *XbaI* for *TRXh3*). Recombinant pProExHTb plasmids containing the wanted coding sequences were used to transform competent *E. coli* cells (HB101l^−^ strain) and single *E. coli* colonies were isolated to verify constructs by sequencing. The sequences of the recombinant proteins generated using recombinant DNA techniques in this study can be found in Supplementary Figure S1.

### 4.3. Production, Purification and Quantification of Recombinant Enzymes

Expression of GAPC1, GRXC1, NTRA and TRX*h*1–*h*5 in *E. coli* was achieved by growing cells in Luria–Bertani broth medium at 37 °C to an absorbance of 0.5 at 600 nm. Recombinant protein production was induced with isopropyl β-d-1-thiogalactopyranoside (IPTG). Induction conditions (IPTG concentration, culture time and temperature), were selected for the various recombinant proteins. For GAPC1, cells were induced across 16 h at 17 °C with 0.6 mM IPTG. Cultures were induced across 4 h at 37 °C with 0.6 mM for the expression of GRXC1 and TRX*h*2–*h*5 and with 1 mM IPTG for the expression of TRX*h*1 and NTRA. After induction, cells were collected via centrifugation (2000× *g*) for 20 min at room temperature (RT). The pellets were frozen and kept at −80 °C until used. Poly His-tagged proteins were purified via affinity chromatography on Ni-NTA resin columns under native conditions according to the manufacturer’s instructions (Invitrogen Canada, Burlington, ON, Canada). Eluted fractions containing recombinant proteins were pooled and dialyzed against a 50 mM Tris-HCl buffer, pH 7.5 containing 1 mM MgCl_2_ and 1 mM DTT. The Bradford method was used to measure protein concentration using bovine serum albumin as a standard [78]. Following purification, enzymes were stored at −20 °C in 50% (*v*/*v*) glycerol. The purity of the preparations was analyzed via SDS-PAGE on polyacrylamide gels (12% (*w*/*v*) for GAPC1 and NTRA and 15% (*w*/*v*) for GRXC1 and TRX*h*1*–h*5). The low-range protein molecular weight marker used for the analysis of TRX*h*1–*h*5 was from Bio-Rad (Saint-Laurent, QC, Canada, catalog number 161-0304). The other analyses were conducted with a wide-range protein molecular weight marker from Bio Basic (Markham, ON, Canada, catalog number BSM0431).

### 4.4. Redox Treatments of Proteins

All experiments were carried out in low-retention tubes. Before experiments, recombinant GAPC1 was aliquoted in 120 µL GAPC1 dilution buffer (75 µM final concentration) containing 50 mM Tris-HCl pH 7.8, 4 mM MgCl_2_ and 30% (*v*/*v*) glycerol. The protein was reduced with 10 mM DTT for 15 min at 4 °C and then desalted using a NAP-5 column following the manufacturer’s instructions (Cytiva, Vancouver, BC, Canada) to remove DTT. Reduced GAPC1 was then quantified using the Bradford method and used for different redox treatments. For the oxidative treatments, GAPC1 (60.15 nM final concentration) was incubated for different lengths of time at 4 °C with 5 µM H_2_O_2_. For treatments with SLG, unless otherwise stated, GAPC1 was incubated with SLG in a GAPC1/SLG molar ratio of 1/2000 for 10 min at room temperature. Reactivation of GAPC1 activity was tested with recombinant GRXC1 or TRXs. For the GRXC1- and TRXs-mediated reactivations, 1.5 µM GAPC1 was treated at room temperature with 3 mM SLG (GAPC1/SLG molar ratio of 1/2000) in 100 mM Tris-HCl pH 7.8. After 10 min incubation, the samples were diluted to obtain a GAPC1 concentration of 0.625 µM. For GRXC1 reactivation, the enzyme was further incubated for 40 min at room temperature in 100 mM Tris-HCl pH 7.8 with the GRS containing 0.2 mM NADPH, 2 mM GSH and 6 µg/mL commercial glutathione reductase in the presence or absence of 15 µM GRXC1 and in a final volume of 150 µL. For reactivation with TRXs, GAPC1 was incubated for 30 min with the TRX recycling system (TRS) containing 0.2 mM NADPH and 0.22 µM NTRA in the presence or absence of 20 µM TRX in a final volume of 150 µL. In parallel with the different enzymatic reactivation reactions, GAPC1 samples were also reactivated with 10 mM DTT.

### 4.5. Enzyme Activity Measurements

Before their use in the experiments described in this study, we verified that the various recombinant proteins were active using spectrophotometric assays performed on a VERSAmax microplate reader (Molecular Devices, San Diego, CA, USA). GRXC1 was assayed using a previously described GSH-Disulfide transhydrogenase activity assay [16]. TRX activity was measured with an insulin precipitation assay [79], in which solution turbidity was measured spectrophotometrically at 650 nm and 30 °C for 60 min. For this, commercial bovine insulin was diluted to 2 mg/mL in sterile milli-Q water adjusted to a pH of 2.2 with HCl and the pH of the solution was readjusted at 7.5–8.0 with 0.1 N NaOH before use. The activity of the five recombinant TRXs (2 µM in the assay mixture) was measured in a buffer containing 0.1 M Tris-HCl pH 8.0, 2 mM EDTA, 0.13 mM bovine insulin and 0.33 mM DTT in a final volume of 200 µL. NTRA activity was assayed following the oxidation of NADPH at 340 nm using the TRX-NTR system in the presence of insulin. The assay buffer contained 0.1 M Tris-HCl pH 8.0, 2 mM EDTA, 0.1 mM NADPH, 2 µM TRX, 130 µM bovine insulin (prepared as above) and recombinant NTRA [80]. The decrease in absorbance was measured for 30 min at 30 °C. For measuring GAPC1 activity, a coupled enzyme assay was used [81]. The decrease in absorbance was measured at 340 nm and 30 °C.

### 4.6. Sample Preparation and nanoLC-MS/MS Proteomic Analysis

Recombinant GAPC1 was reduced by incubation with 10 mM DTT for 15 min at 4 °C, desalted and quantified as described above. Reduced GAPC1 (0.5 µM) was then treated for 10 min at room temperature with 1 mM SLG or left untreated (control). Samples containing 100 pmol GAPC1 were then either dialyzed overnight at room temperature in a buffer containing 100 mM NH_4_HCO_3_ or reduced with 30 mM DTT at 56 °C for 45 min, alkylated in the dark with 70 mM iodoacetamide at room temperature for 30 min and then dialyzed in 100 mM NH_4_HCO_3_ buffer overnight at room temperature. After dialysis, the samples were digested overnight at 37 °C in a buffer containing 100 mM NH_4_HCO_3_, 10% (*v*/*v*) acetonitrile, 2.5 mM CaCl_2_ and trypsin (modified, sequencing grade, Promega, Madison, WI, USA) using a trypsin/protein ratio of 1:50. The digested samples were dried down and rehydrated in 15 µL of buffer containing 2% (*v*/*v*) acetonitrile, 1% (*v*/*v*) formic acid, vortexed for 30 min, and centrifuged at 20,000× *g* for 30 min, and aliquots were used for nanoLC-MS/MS analysis. The automated nanoLC-MS/MS analysis of peptide digests with 30 pmol of the initial protein sample at the final concentration of 6.6 pmol/µL was performed using Q Exactive™ Hybrid Quadrupole-Orbitrap mass spectrometer equipped with a nanospray Flex NG source, connected on-line with an EASY-nLC 1000 system (Thermo Fisher Scientific, San Jose, CA, USA). Five microliter aliquots were separated by on-line liquid chromatography using an in-house packed reversed-phase nano-column Luna C18 (75 µm ID, 360 µm OD, 18 cm length, 5 µm, 100 Å, Phenomenex Inc., Torrance, CA, USA), at a flow rate of 300 nL/min. The column was evaluated for quality control with 200 ng Pierce HeLa Protein Digest Standard (Thermo Fisher Scientific, San Jose, CA, USA) with over 2800 identified unique proteins. The peptide elution was performed using a linear 135 min gradient of 2–20% (*v*/*v*) ACN for 105 min, followed by a gradient of 20–32% (*v*/*v*) ACN for 20 min, 32–95% (*v*/*v*) ACN for 2 min, and 99.9% (*v*/*v*) ACN for 8 min in 0.1% (*v*/*v*) formic acid.

Data-dependent acquisition in the Q Exactive instrument proceeded with nano-electrospray voltage 2.1 kV, capillary temperature 275 °C, in 1.8 s scan cycles, starting by a single full scan MS at *m*/*z* range 400–1800 in a profile mode, resolution 70,000 full width at half-maximum (FWHM) at 200 *m*/*z*, one microscan with maximum inject time 100 ms. Survey spectra were followed by MS/MS fragmentation of 12 most intense ions’ charge state 2–7, selected from the full scan MS for HCD at normalized collision energy of 27% with profile mode detection, the Orbitrap resolution 17,500 FWHM at 200 *m*/*z*, 80 *m*/*z* fixed first mass scan range to 2000 *m*/*z*, 1 microscan, and 120 ms maximum injection time. The automatic gain control target values for full MS and MS/MS were 3 × 10^6^ and 2 × 10^5^, respectively. Dynamic exclusion of 15 s was employed as well as rejection of charge state unassigned, 8, >8 with precursors isolated at 2 *m*/*z* width.

### 4.7. Proteomics Data Analysis and Interpretation

Peptide HCD fragmentation spectra were searched using Mascot Server v. 2.8.3 (Matrix Science, London, UK) against the Araport11 (48,270 sequences; 20,842,213 residues) Arabidopsis protein database using a general search with specified modifications, followed by the Error Tolerant Search to allow for protein modification screening and finally Mascot searches with crosslinking analysis and mapping of the disulfide bridges. The Mascot MS/MS Ion Search parameters were as follows: (1) tryptic digest with maximum two missed cleavage; (2) monoisotopic peptide masses were used; (3) the peptide mass tolerance was kept at 20 ppm; and the fragment ions mass tolerance was set at 20 ppm; (4) variable modifications Glutathione (C), Carbamidomethyl (C), Oxidation (M), Deamidation (NQ), and Dehydro (C) were used; (5) peptide charge state +1, +2 and +3 for nanoLC-MS/MS spectra. The Error Tolerant Search was conducted with two variable modifications allowed: Glutathione (C) and LactoylLys (K). Crosslinking Search for a disulfide bridge mapping in GAPC1 was conducted with variable modifications: Oxidation (M), LactoylLys (K). SIM-XL software (V 1.5.7.1) (http://patternlabforproteomics.org/sim-xl, (accessed on 25 April 2025) was also used for the identification of Xlinked Disulfides [82]. Spectra with modifications were verified manually using the GPMAW 12.11 (Lighthouse Data, Odense, Denmark) software, Freestyle v1.5 and Qual Browser Xcalibur v4.2.47 software programs (Thermo Fisher Scientific, San Jose, CA, USA) for visualizing and analyzing chromatograms and spectra.

### 4.8. Structural Prediction of GAPC1 Structures

The tetrameric structures of GAPC1 apo, GAPC1 glutathionylated on Cys^156^, GAPC1 glutathionylated on both Cys^156^ and Cys^160^ or GAPC1 lactoylated on individual Lys^76,190,198,219,255,310^ were predicted using AlphaFold v3.0.0 [83] installed on the Narval cluster of the Digital Research Alliance of Canada. AlphaFold3 predictions contained an extraneous OXT atom on the lactoyl groups that was manually removed prior to analysis. Figures were generated using PyMOL v3.1.0.

### 4.9. Statistical Analyses

All experiments were conducted with at least three independent experimental replicates and each individual experiment included three technical replicates. The reported data are means ± SD, which were analyzed using the Student’s *t*-test with *p* < 0.05 considered as a significant difference. Statistical analyses were performed using Microsoft Excel.

## Data Availability

The biological material and the data presented in this study are available from the corresponding author upon reasonable request.

## Supplementary Materials

The following supporting information can be downloaded at: https://www.mdpi.com/article/10.3390/ijms26199673/s1.

## Abbreviations

The following abbreviations are used in this manuscript:

GAPC1	cytosolic glyceraldehyde-3-phosphate dehydrogenase C1
ROS	reactive oxygen species
MG	methylglyoxal
PTM	post-translational protein modification
GSH	reduced glutathione
GSSG	oxidized glutathione
SLG	*S*-D-lactoylglutathione
GS^−^	glutathione thiolate
GLO1	glyoxalase I
GLO2	glyoxalase II
GAPDH	Glyceraldehyde-3-phosphate dehydrogenase
Arabidopsis	*Arabidopsis thaliana*
MDH	malate dehydrogenase
DTT	dithiotreitol
IPTG	isopropyl β-D-1-thiogalactopyranoside
GRX	Glutaredoxin
TRX	thioredoxin
GR	NADPH-dependent glutathione reductase
NTR	NADPH-dependent thioredoxin reductase
nanoLC-MS/MS	nano liquid chromatography-tandem mass spectrometry
FWHM	full width at half-maximum
HCD	Higher-energy Collision Dissociation
Dha	dehydroalanine
GRS	glutaredoxin recycling system
TRS	thioredoxin recycling system

## References

[1] Mareri L., Parrotta L., Cai G. (2022). Environmental Stress and Plants. Int. J. Mol. Sci..

[2] Iqbal Z., Iqbal M.S., Hashem A., Abd_Allah E.F., Ansari M.I. (2021). Plant Defense Responses to Biotic Stress and Its Interplay with Fluctuating Dark/Light Conditions. Front. Plant Sci..

[3] Zhang H., Zhu J., Gong Z., Zhu J.-K. (2022). Abiotic stress responses in plants. Nat. Rev. Genet..

[4] Zhang Y., Xu J., Li R., Ge Y., Li Y., Li R. (2023). Plants’ Response to Abiotic Stress: Mechanisms and Strategies. Int. J. Mol. Sci..

[5] Dorion S., Ouellet J.C., Rivoal J. (2021). Glutathione metabolism in plants under stress: Beyond reactive oxygen species detoxification. Metabolites.

[6] Foyer C.H., Kunert K. (2024). The ascorbate–glutathione cycle coming of age. J. Exp. Bot..

[7] Mostofa M.G., Ghosh A., Li Z.G., Siddiqui M.N., Fujita M., Tran L.S.P. (2018). Methylglyoxal-A signaling molecule in plant abiotic stress responses. Free Radic. Biol. Med..

[8] Singh D. (2022). Juggling with reactive oxygen species and antioxidant defense system–A coping mechanism under salt stress. Plant Stress..

[9] Boutin C., Clément C., Rivoal J. (2024). Post-Translational Modifications to Cysteine Residues in Plant Proteins and Their Impact on the Regulation of Metabolism and Signal Transduction. Int. J. Mol. Sci..

[10] Dalle-Donne I., Rossi R., Colombo G., Giustarini D., Milzani A. (2009). Protein S-glutathionylation: A regulatory device from bacteria to humans. Trends Biochem. Sci..

[11] Zhang J., Ye Z.-w., Singh S., Townsend D.M., Tew K.D. (2018). An evolving understanding of the *S*-glutathionylation cycle in pathways of redox regulation. Free Radic. Biol. Med..

[12] Ghezzi P. (2013). Protein glutathionylation in health and disease. Biochim. Et Biophys. Acta (BBA)-General. Subj..

[13] Lermant A., Murdoch C.E. (2019). Cysteine Glutathionylation Acts as a Redox Switch in Endothelial Cells. Antioxidants.

[14] van der Linde K., Gutsche N., Leffers H.-M., Lindermayr C., Müller B., Holtgrefe S., Scheibe R. (2011). Regulation of plant cytosolic aldolase functions by redox modifications. Plant Physiol. Biochem..

[15] Bedhomme M., Adamo M., Marchand C.H., Couturier J., Rouhier N., Lemaire S.D., Zaffagnini M., Trost P. (2012). Glutathionylation of cytosolic glyceraldehyde-3-phosphate dehydrogenase from the model plant *Arabidopsis thaliana* is reversed by both glutaredoxins and thioredoxins in vitro. Biochem. J..

[16] Dumont S., Bykova N.V., Pelletier G., Dorion S., Rivoal J. (2016). Cytosolic triosephosphate isomerase from *Arabidopsis thaliana* is reversibly modified by glutathione on cysteines 127 and 218. Front. Plant Sci..

[17] Bender K.W., Wang X., Cheng G.B., Kim H.S., Zielinski R.E., Huber S.C. (2015). Glutaredoxin AtGRXC2 catalyses inhibitory glutathionylation of *Arabidopsis* BRI1-associated receptor-like kinase 1 (BAK1) in vitro. Biochem. J..

[18] Chan K.X., Mabbitt P.D., Phua S.Y., Mueller J.W., Nisar N., Gigolashvili T., Stroeher E., Grassl J., Arlt W., Estavillo G.M. (2016). Sensing and signaling of oxidative stress in chloroplasts by inactivation of the SAL1 phosphoadenosine phosphatase. Proc. Natl. Acad. Sci. USA.

[19] Gurrieri L., Distefano L., Pirone C., Horrer D., Seung D., Zaffagnini M., Rouhier N., Trost P., Santelia D., Sparla F. (2019). The thioredoxin-regulated α-amylase 3 of *Arabidopsis thaliana* is a target of *S*-glutathionylation. Front. Plant Sci..

[20] Thornalley P.J. (2008). Protein and nucleotide damage by glyoxal and methylglyoxal in physiological systems-role in ageing and disease. Drug Metabol. Drug Interact..

[21] Thornalley P.J. (1990). The glyoxalase system-New developments towards functional-characterization of a metabolic pathway fundamental to biological life. Biochem. J..

[22] Deswal R., Chakaravarty T.N., Sopory S.K. (1993). The glyoxalase system in higher plants: Regulation in growth and differentiation. Biochem. Soc. Trans..

[23] Maiti M.K., Krishnasamy S., Owen H.A., Makaroff C.A. (1997). Molecular characterization of glyoxalase II from Arabidopsis thaliana. Plant Mol. Biol..

[24] Engqvist M., Drincovich M.F., Flugge U.I., Maurino V.G. (2009). Two D-2-hydroxy-acid dehydrogenases in *Arabidopsis thaliana* with catalytic capacities to participate in the last reactions of the methylglyoxal and beta-oxidation pathways. J. Biol. Chem..

[25] Gaffney D.O., Jennings E.Q., Anderson C.C., Marentette J.O., Shi T., Schou Oxvig A.-M., Streeter M.D., Johannsen M., Spiegel D.A., Chapman E. (2020). Non-enzymatic Lysine Lactoylation of Glycolytic Enzymes. Cell Chem. Biol..

[26] Trujillo M.N., Jennings E.Q., Hoffman E.A., Zhang H., Phoebe A.M., Mastin G.E., Kitamura N., Reisz J.A., Megill E., Kantner D. (2023). Lactoylglutathione promotes inflammatory signaling in macrophages through histone lactoylation. Mol. Metab..

[27] Zhang D., Tang Z., Huang H., Zhou G., Cui C., Weng Y., Liu W., Kim S., Lee S., Perez-Neut M. (2019). Metabolic regulation of gene expression by histone lactylation. Nature.

[28] Zhao W., Xin J., Yu X., Li Z., Li N. (2025). Recent advances of lysine lactylation in prokaryotes and eukaryotes. Front. Mol. Biosci..

[29] Wang Z., Hao D., Zhao S., Zhang Z., Zeng Z., Wang X. (2023). Lactate and Lactylation: Clinical Applications of Routine Carbon Source and Novel Modification in Human Diseases. Mol. Cell. Proteom..

[30] Wan N., Wang N., Yu S., Zhang H., Tang S., Wang D., Lu W., Li H., Delafield D.G., Kong Y. (2022). Cyclic immonium ion of lactyllysine reveals widespread lactylation in the human proteome. Nat. Methods.

[31] Ercolani L., Scirè A., Galeazzi R., Massaccesi L., Cianfruglia L., Amici A., Piva F., Urbanelli L., Emiliani C., Principato G. (2016). A possible S-glutathionylation of specific proteins by glyoxalase II: An in vitro and in silico study. Cell Biochem. Funct..

[32] Zaffagnini M., Fermani S., Costa A., Lemaire S.D., Trost P. (2013). Plant cytoplasmic GAPDH: Redox post-translational modifications and moonlighting properties. Front. Plant Sci..

[33] Rius S.P., Casati P., Iglesias A.A., Gomez-Casati D.F. (2008). Characterization of Arabidopsis lines deficient in GAPC-1, a cytosolic NAD-dependent glyceraldehyde-3-phosphate dehydrogenase. Plant Physiol..

[34] Cao Y., Hong J., Wang H., Lin M., Cai Y., Liao L., Li X., Han Y. (2025). Beyond glycolysis: Multifunctional roles of glyceraldehyde-3-phosphate dehydrogenases in plants. Hortic. Res..

[35] Holtgrefe S., Gohlke J., Starmann J., Druce S., Klocke S., Altmann B., Wojtera J., Lindermayr C., Scheibe R. (2008). Regulation of plant cytosolic glyceraldehyde 3-phosphate dehydrogenase isoforms by thiol modifications. Physiol. Plant..

[36] Dumont S., Rivoal J. (2019). Consequences of oxidative stress on plant glycolytic and respiratory metabolism. Front. Plant Sci..

[37] Zaffagnini M., Marchand C.H., Malferrari M., Murail S., Bonacchi S., Genovese D., Montalti M., Venturoli G., Falini G., Baaden M. (2019). Glutathionylation primes soluble glyceraldehyde-3-phosphate dehydrogenase for late collapse into insoluble aggregates. Proc. Natl. Acad. Sci. USA.

[38] Michalski A., Neuhauser N., Cox J., Mann M. (2012). A Systematic Investigation into the Nature of Tryptic HCD Spectra. J. Proteome Res..

[39] Saminathan I.S., Wang X.S., Guo Y., Krakovska O., Voisin S., Hopkinson A.C., Siu K.W.M. (2010). The extent and effects of peptide sequence scrambling via formation of macrocyclic b ions in model proteins. J. Am. Soc. Mass. Spectrom..

[40] Su D., Gaffrey M.J., Guo J., Hatchell K.E., Chu R.K., Clauss T.R.W., Aldrich J.T., Wu S., Purvine S., Camp D.G. (2014). Proteomic identification and quantification of S-glutathionylation in mouse macrophages using resin-assisted enrichment and isobaric labeling. Free Radic. Biol. Med..

[41] Li X., Day N.J., Feng S., Gaffrey M.J., Lin T.-D., Paurus V.L., Monroe M.E., Moore R.J., Yang B., Xian M. (2021). Mass spectrometry-based direct detection of multiple types of protein thiol modifications in pancreatic beta cells under endoplasmic reticulum stress. Redox Biol..

[42] Crooks G.E., Hon G., Chandonia J.M., Brenner S.E. (2004). WebLogo: A sequence logo generator. Genome Res..

[43] Huang J.J., Willems P., Van Breusegem F., Messens J. (2018). Pathways crossing mammalian and plant sulfenomic landscapes. Free Radic. Biol. Med..

[44] Ferrer-Sueta G., Manta B., Botti H., Radi R., Trujillo M., Denicola A. (2011). Factors Affecting Protein Thiol Reactivity and Specificity in Peroxide Reduction. Chem. Res. Toxicol..

[45] Zaffagnini M., Fermani S., Calvaresi M., Orrù R., Iommarini L., Sparla F., Falini G., Bottoni A., Trost P. (2016). Tuning Cysteine Reactivity and Sulfenic Acid Stability by Protein Microenvironment in Glyceraldehyde-3-Phosphate Dehydrogenases of Arabidopsis thaliana. Antioxid. Redox Signal..

[46] Zaffagnini M., Michelet L., Marchand C., Sparla F., Decottignies P., Le Marechal P., Miginiac-Maslow M., Noctor G., Trost P., Lemaire S.D. (2007). The thioredoxin-independent isoform of chloroplastic glyceraldehyde-3-phosphate dehydrogenase is selectively regulated by glutathionylation. FEBS J..

[47] Kyte J. (2024). Mechanisms for reactions, nucleophilic substitution at an acyl group. Mechanism in Protein Chemistry.

[48] Wagner G.R., Payne R.M. (2013). Widespread and enzyme-independent *N*^ϵ^-acetylation and *N*^ϵ^-succinylation of proteins in the chemical conditions of the mitochondrial matrix. J. Biol. Chem..

[49] Grek C.L., Zhang J., Manevich Y., Townsend D.M., Tew K.D. (2013). Causes and consequences of cysteine S-glutathionylation. J. Biol. Chem..

[50] Matsui R., Ferran B., Oh A., Croteau D., Shao D., Han J., Pimentel D.R., Bachschmid M.M. (2020). Redox Regulation via Glutaredoxin-1 and Protein S-Glutathionylation. Antioxid. Redox Signal..

[51] Caccuri A.M., Antonini G., Board P.G., Parker M.W., Nicotra M., Lo Bello M., Federici G., Ricci G. (1999). Proton release on binding of glutathione to alpha, Mu and Delta class glutathione transferases. Biochem. J..

[52] Orozco M., Vega C., Parraga A., GarciaSaez I., Coll M., Walsh S., Mantle T.J., Luque F.J. (1997). On the reaction mechanism of class pi glutathione S-transferase. Proteins-Struct. Funct. Genet..

[53] Townsend D.M., Manevich Y., He L., Hutchens S., Pazoles C.J., Tew K.D. (2009). Novel role for glutathione S-transferase Pi regulator of protein s-glutathionylation following oxidative and nitrosative stress. J. Biol. Chem..

[54] Zhang H., Zhao Y., Zhou D.X. (2017). Rice NAD+-dependent histone deacetylase OsSRT1 represses glycolysis and regulates the moonlighting function of GAPDH as a transcriptional activator of glycolytic genes. Nucleic Acids Res..

[55] Liu Z., Song J., Miao W., Yang B., Zhang Z., Chen W., Tan F., Suo H., Dai X., Zou X. (2021). Comprehensive Proteome and Lysine Acetylome Analysis Reveals the Widespread Involvement of Acetylation in Cold Resistance of Pepper (*Capsicum annuum* L.). Front. Plant Sci..

[56] Finkemeier I., Laxa M., Miguet L., Howden A.J.M., Sweetlove L.J. (2011). Proteins of Diverse Function and Subcellular Location Are Lysine Acetylated in Arabidopsis. Plant Physiol..

[57] Yang S.S., Zhai Q.H. (2017). Cytosolic GAPDH: A key mediator in redox signal transduction in plants. Biol. Plant..

[58] Guo L., Devaiah S.P., Narasimhan R., Pan X., Zhang Y., Zhang W., Wang X. (2012). Cytosolic Glyceraldehyde-3-Phosphate Dehydrogenases Interact with Phospholipase D+¦ to Transduce Hydrogen Peroxide Signals in the Arabidopsis Response to Stress. Plant Cell.

[59] Henry E., Fung N., Liu J., Drakakaki G., Coaker G. (2015). Beyond glycolysis: GAPDHs are multi-functional enzymes involved in regulation of ROS, autophagy, and plant immune responses. PLoS Genet..

[60] Kim S.-C., Guo L., Wang X. (2020). Nuclear moonlighting of cytosolic glyceraldehyde-3-phosphate dehydrogenase regulates Arabidopsis response to heat stress. Nat. Commun..

[61] Wegener M., Persicke M., Dietz K.-J. (2023). Reprogramming the translatome during daily light transitions as affected by cytosolic glyceraldehyde-3-phosphate dehydrogenases GAPC1/C2. J. Exp. Bot..

[62] Peralta D.A., Araya A., Busi M.V., Gomez-Casati D.F. (2016). The E3 ubiquitin-ligase SEVEN IN ABSENTIA like 7 mono-ubiquitinates glyceraldehyde-3-phosphate dehydrogenase 1 isoform in vitro and is required for its nuclear localization in Arabidopsis thaliana. Int. J. Biochem. Cell Biol..

[63] Geigenberger P., Thormählen I., Daloso D.M., Fernie A.R. (2017). The Unprecedented Versatility of the Plant Thioredoxin System. Trends Plant Sci..

[64] Mondal S., Singh S.P. (2022). New insights on thioredoxins (Trxs) and glutaredoxins (Grxs) by in silico amino acid sequence, phylogenetic and comparative structural analyses in organisms of three domains of life. Heliyon.

[65] Chibani K., Pucker B., Dietz K.-J., Cavanagh A. (2021). Genome-wide analysis and transcriptional regulation of the typical and atypical thioredoxins in *Arabidopsis thaliana*. FEBS Lett..

[66] Riondet C., Desouris J.P., Guilleminot-Montoya J., Chartier Y., Meyer Y., Reichheld J.-P. (2012). A dicotyledon-specific glutaredoxin GRXC1 family with dimer-dependent redox regulation is functionally redundant with GRXC2. Plant Cell Environ..

[67] Bodnar Y., Gellert M., Hossain F.M., Lillig C.H. (2023). Breakdown of *Arabidopsis thaliana* thioredoxins and glutaredoxins based on electrostatic similarity-Leads to common and unique interaction partners and functions. PLoS ONE.

[68] Zaffagnini M., Michelet L., Massot V., Trost P., Lemaire S.D. (2008). Biochemical Characterization of Glutaredoxins from Chlamydomonas reinhardtii Reveals the Unique Properties of a Chloroplastic CGFS-type Glutaredoxin*. J. Biol. Chem..

[69] Singla-Pareek S.L., Kaur C., Kumar B., Pareek A., Sopory S.K. (2020). Reassessing plant glyoxalases: Large family and expanding functions. New Phytol..

[70] Yadav S.K., Singla-Pareek S.L., Ray M., Reddy M.K., Sopory S.K. (2005). Methylglyoxal levels in plants under salinity stress are dependent on glyoxalase I and glutathione. Biochem. Biophys. Res. Commun..

[71] Gupta B.K., Sahoo K.K., Ghosh A., Tripathi A.K., Anwar K., Das P., Singh A.K., Pareek A., Sopory S.K., Singla-Pareek S.L. (2018). Manipulation of glyoxalase pathway confers tolerance to multiple stresses in rice. Plant Cell Environ..

[72] Kaur C., Singla-Pareek S.L., Sopory S.K. (2014). Glyoxalase and methylglyoxal as biomarkers for plant stress tolerance. Crit. Rev. Plant Sci..

[73] Hossain M.A., Hossain M.Z., Fujita M. (2009). Stress-induced changes of methylglyoxal level and glyoxalase I activity in pumpkin seedlings and cDNA cloning of glyoxalase I gene. Aust. J. Crop Sci..

[74] Chen Z.Y., Brown R.L., Damann K.E., Cleveland T.E. (2004). Identification of a maize kernel stress-related protein and its effect on aflatoxin accumulation. Phytopathology.

[75] Moreno J.C., Rojas B.E., Vicente R., Gorka M., Matz T., Chodasiewicz M., Peralta-Ariza J.S., Zhang Y., Alseekh S., Childs D. (2021). Tyr-Asp inhibition of glyceraldehyde 3-phosphate dehydrogenase affects plant redox metabolism. EMBO J..

[76] Alonso J.M., Stepanova A.N., Leisse T.J., Kim C.J., Chen H., Shinn P., Stevenson D.K., Zimmerman J., Barajas P., Cheuk R. (2003). Genome-wide insertional mutagenesis of *Arabidopsis thaliana*. Science.

[77] Green M.R., Sambrook J. (2012). Molecular Cloning: A Laboratory Manual.

[78] Bradford M.M. (1976). A rapid and sensitive method for the quantitation of microgram quantities of protein utilizing the principle of protein-dye binding. Anal. Biochem..

[79] Holmgren A. (1979). Thioredoxin catalyzes the reduction of insulin disulfides by dithiothreitol and dihydrolipoamide. J. Biol. Chem..

[80] Dai C., Wang M.H. (2011). Isolation and characterization of thioredoxin and NADPH-dependent thioredoxin reductase from tomato (*Solanum lycopersicum*). BMB Rep..

[81] Claeyssen E., Dorion S., Clendenning A., He J.Z., Wally O., Chen J., Auslender E.L., Moisan M.-C., Jolicoeur M., Rivoal J. (2013). The futile cycling of hexose phosphates could account for the fact that hexokinase exerts a high control on glucose phosphorylation but not on glycolytic rate in transgenic potato (*Solanum tuberosum*) Roots. PLoS ONE.

[82] Lima D.B., de Lima T.B., Balbuena T.S., Neves-Ferreira A.G.C., Barbosa V.C., Gozzo F.C., Carvalho P.C. (2015). SIM-XL: A powerful and user-friendly tool for peptide cross-linking analysis. J. Proteom..

[83] Abramson J., Adler J., Dunger J., Evans R., Green T., Pritzel A., Ronneberger O., Willmore L., Ballard A.J., Bambrick J. (2024). Accurate structure prediction of biomolecular interactions with AlphaFold 3. Nature.

