# Supplementary material for: Implication of S-d-Lactoylglutathione in the Spontaneous Cysteine S-Glutathionylation and Lysine N-Lactoylation of Arabidopsis thaliana NAD-Dependent Glyceraldehyde-3-Phosphate Dehydrogenase
Source: Int J Mol Sci. 2025 Oct 3;26(19):9673. doi: 10.3390/ijms26199673 (PMC12524660; doi:10.3390/ijms26199673)
Supplement: Supplementary file 1 [file ijms-26-09673-s001.zip › ijms-3872620-supplementary.pdf]

Supplementary material

Implication of *S*-D-lactoylglutathione in the spontaneous cysteine *S*-glutathionylation and lysine N-lactoylation of *Arabidopsis thaliana* NAD-dependent Glyceraldehyde-3-Phosphate Dehydrogenase

Authors: [1]Camille Clément, [1]Sonia Dorion, [2]Natalia Bykova, [1]Vincent Fetterley, [1]Elvis Branchini, [1]Charlie Boutin, [3]Laurent Cappadocia, [1]Jean Rivoal

[1]Institut de Recherche en Biologie Végétale, Université de Montréal, Montréal, Québec, Canada

[2]Morden Research and Development Centre, Agriculture and Agri-Food Canada, Morden, MB, Canada

[3]Département de Chimie, Université du Québec à Montréal, Montréal, Québec, Canada

Correspondance to: Jean Rivoal: jean.rivoal@umontreal.ca

Supplementary Table 1. List of the gene IDs, gene names, ABRC stock numbers and PCR primers that were used to generate the constructs coding for recombinant proteins in this study.

Gene ID	Name (abbrevation)	ABRC Stock number	Forward (F) and reverse (R) primers (5' to 3')
At3g04120	glyceraldehyde-3-phosphate dehydrogenase C1 (GAPC1)	U13633	F: 5' ATGGCTGACAAGAAGATTAGG 3'<br>R: 5' TGTTAGCAGCCGGATCTTCTA 3'
At5g39950	thioredoxin *h*2 (TRX*h*2)	U13070	F: 5' ATGGGAGGAGCTTTATCAACT 3'<br>R: 5' TGTTAGCAGCCGGATCTTCTA 3'
At5g42980	thioredoxin *h*3 (TRX*h*3)	U15950	F: 5' ATGGCCGCAGAAGGAGAAGTT 3'<br>R: 5' GAGATCTAGAGAGCTGCCAGGAAACAGCTA 3'
At1g19730	thioredoxin *h*4 (TRX*h*4)	U61017	F: 5' ATGGCGGCAGAAGAGGGTC 3'<br>R: 5' TGGCTGGCAACTAGAAGGCAC 3'
At1g45145	thioredoxin *h*5 (TRX*h*5)	U09186	F: 5' ATGGCCGGTGAAGGAGAAGTG 3'<br>R: 5' TGGCTGGCAACTAGAAGGCAC 3'

Supplementary Figure S1

A (6xHis)GAPC1:

MSYYHHHHHHDYDIPTTENLYFQGMADKKIRIGINGFGRIGRLVARVVLQRDDVELVAVNDPFITTEYMTYMFKYDSVHGQWK
HNELKIKDEKTLLFGEKPVTVFGIRNPEDIPWAEAGADYVVESTGVFTDKDKAAAHLKGGAKKVVISAPSKDAPMFVVGVNEH
EYKSDLDIVSNASCTTNCLAPLAKVINDRFGIVEGLMTTVHSITATQKTVDGPSMKDWRGGRAASFNIIPSSTGAAKAVGKVL
PALNGKLTGMSFRVPTVDVSVVDLTVRLEKAATYDEIKKAIKEESEGKLKGILGYTEDDVVSTDFVGDNRSSIFDAKAGIALS
DKFVKLVSWYDNEWGYSSRVVDLIVHMSKA

B (10xHis)TRX*h*1:

MGHHHHHHHHHHSSGHIDDDDKHMASEEGQVIACHTVETWNEQLQKANESKTLVVVDFTASWCGPCRFIAPFFADLAKKLPNV
LFLKVDTDELKSVASDWAIQAMPTFMFLKEGKILDKVVGAKKDELQSTIAKHLA

C (6xHis)TRX*h*2:

MSYYHHHHHHDYDIPTTENLYFQGMGGALSTVFGSGEDATAAGTESEPSRVLKFSSSARWQLHFNEIKESNKLLVVDFSASWC
GPCRMIEPAIHAMADKFNDVDFVKLDVDELPDVAKEFNVTAMPTFVLVKRGKEIERIIGAKKDELEKKVSKLRAMGGALSTVF
GSGEDATAAGTESEPSRVLKFSSSARWQLHFNEIKESNKLLVVDFSASWCGPCRMIEPAIHAMADKFNDVDFVKLDVDELPDV
AKEFNVTAMPTFVLVKRGKEIERIIGAKKDELEKKVSKLRA

D (6xHis)TRX*h*3:

MSYYHHHHHHDYDIPTTENLYFQGMAAEGEVIACHTVEDWTEKLKAANESKKLIVIDFTATWCPPCRFIAPVFADLAKKHLDV
VFFKVDVDELNTVAEEFKVQAMPTFIFMKEGEIKETVVGAAKEEIIANLEKHKTVVAAA

E (6xHis)TRX*h*4:

MSYYHHHHHHDYDIPTTENLYFQGMAAEEGQVIGCHTNDVWTVQLDKAKESNKLIVIDFTASWCPPCRMIAPIFNDLAKKFMS
SAIFFKVDVDELQSVAKEFGVEAMPTFVFIKAGEVVDKLVGANKEDLQAKIVKHTGVTTA

F (6xHis)TRX*h*5:

MSYYHHHHHHDYDIPTTENLYFQGMAGEGEVIACHTLEVWNEKVKDANESKKLIVIDFTASWCPPCRFIAPVFAEMAKKFTNV
VFFKIDVDELQAVAQEFKVEAMPTFVFMKEGNIIDRVVGAAKDEINEKLMKHGGLVASA

G (10xHis)NTRA

MGHHHHHHHHHHSSGHIDDDDKHMETHKTKVCIVGSGPAAHTAAIYASRAELKPLLFEGWMANDIAPGGQLTTTTDVENFPGF
PEGILGIDIVEKFRKQSERFGTTIFTETVNKVDFSSKPFKLFTDSRTVLADSVIISTGAVAKRLSFTGSGEGNGGFWNRGISA
CAVCDGAAPIFRNKPLVVIGGGDSAMEEANFLTKYGSKVYIIHRRDTFRASKIMQQRALSNPKIEVIWNSAVVEAYGDENGRV
LGGLKVKNVVTGDVSDLKVSGLFFAIGHEPATKFLDGQLELDEDGYVVTKPGTTKTSVVGVFAAGDVQDKKYRQAITAAGTGC
MAALDAEHYLQEIGSQEGKSD

Supplementary Figure S1 : Sequences of the recombinant proteins generated for this study. **A**, (6xHis)GAPC1; **B**, (10xHis)TRX*h*1; **C**, (6xHis)TRX*h*2; **D**, (6xHis)TRX*h*3; **E**, (6xHis)TRX*h*4; **F**, (6xHis)TRX*h*5; **G**, (10xHis)NTRA. In each sequence, the N-terminal stretch of amino acid highlighted in bold indicates the sequence derived from the vector.

Supplementary Figure S2

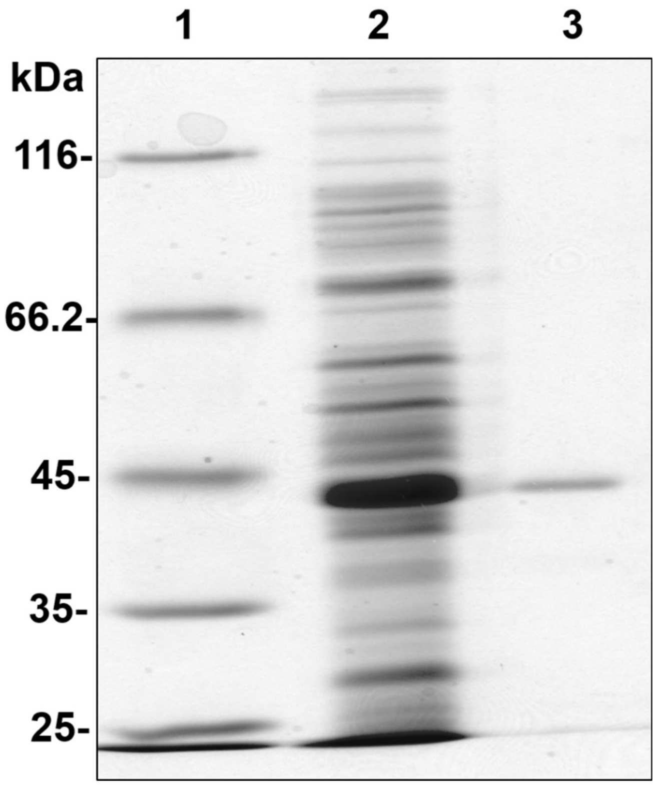

Supplementary Figure S2. SDS-PAGE analysis of the purification of the recombinant *Arabidopsis thaliana* (6xHis)GAPC1. Lane 1, molecular weight markers; lane 2, protein extract of the IPTG-induced *E. coli* culture; lane 3, purified recombinant (6xHis)GAPC1. Numbers on the left side indicate the running position and size in kDa of molecular weight markers.

Supplementary Figure S3

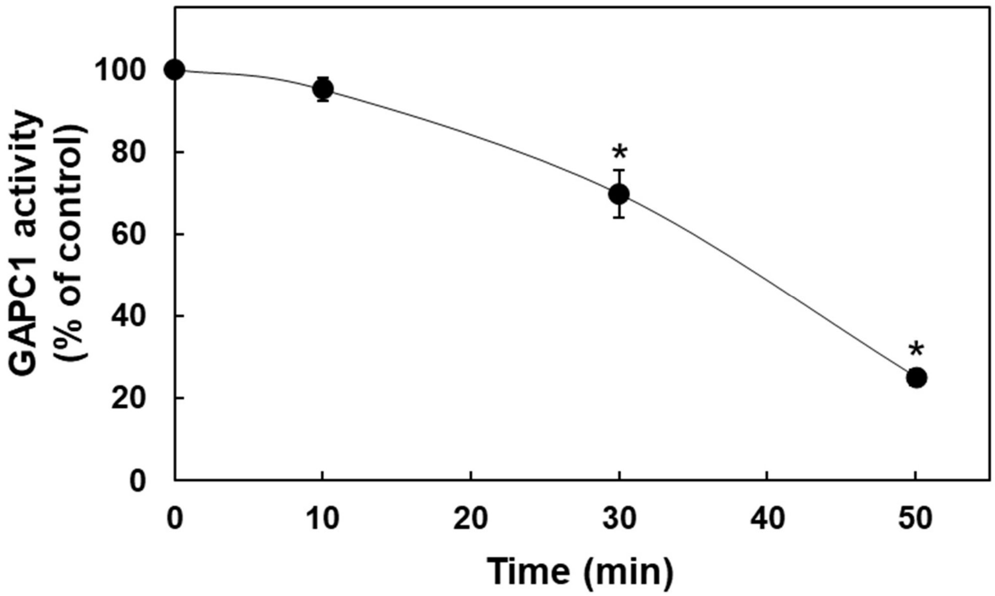

Supplementary Figure S3. Inhibition of GAPC1 activity by H_2O_2. Recombinant GAPC1 was incubated with 5 µM H_2O_2 for up to 50 min. Remaining activity was measured at different time intervals and expressed expressed as % of activity obtained with enzyme incubated for the same time in absence of H_2O_2 (mean ± SD, n = 3). An asterisk (*) marks a statistical difference between a treatment and the time 0 control as determined using a *t*-test (p< 0.05).

Supplementary Figure S4

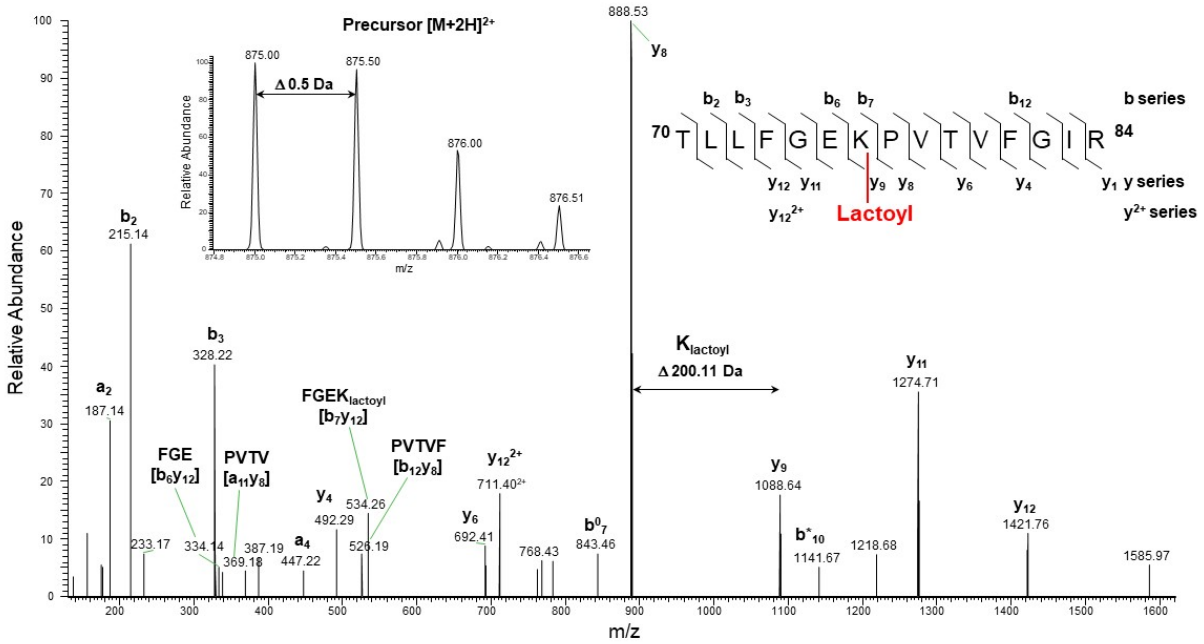

Supplementary Figure S4. NanoLC-MS/MS spectrum of the precursor ion $[M+2H]^{2+}$ at *m/z* 875.00^{2+} fragmented with Higher-energy Collision Dissociation (HCD) identifies an *N*-lactoyl Lys modification site at Lys^{76} residue. Sequence specific b- and y-type backbone fragment ion signals, and a-type ions that derive from the corresponding b-type ion by losing CO, identify the peptide sequence ^{70}TLLFGEKPVTVFGIR^{84} (top right corner). The *N*-lactoyl Lys modification site was assigned by the 200.11 Da mass difference between y_8 and y_9 ions of C-terminal peptide fragments. The peaks denoted b^0 and b^* are the result of water (-18 Da) or ammonia (-17 Da) loss from corresponding ions, respectively. Internal peptide fragments labeled with "by" nomenclature indicate ions formed by double cleavage events in the peptide backbone. The internal fragment ion b_7y_{12}, as well as the sequence-specific y_9, y_{11}, y_{12}, b_7, and b_{12} ions provided high confidence evidence for the assignment of *N*-lactoyl Lys modification site at Lys^{76} residue. The spectral portion of precursor ion MS scan with isotopically resolved peaks indicating the parent ion charge state $[M+2H]^{2+}$ is shown in the insert (top left corner).

Supplementary Figure S5

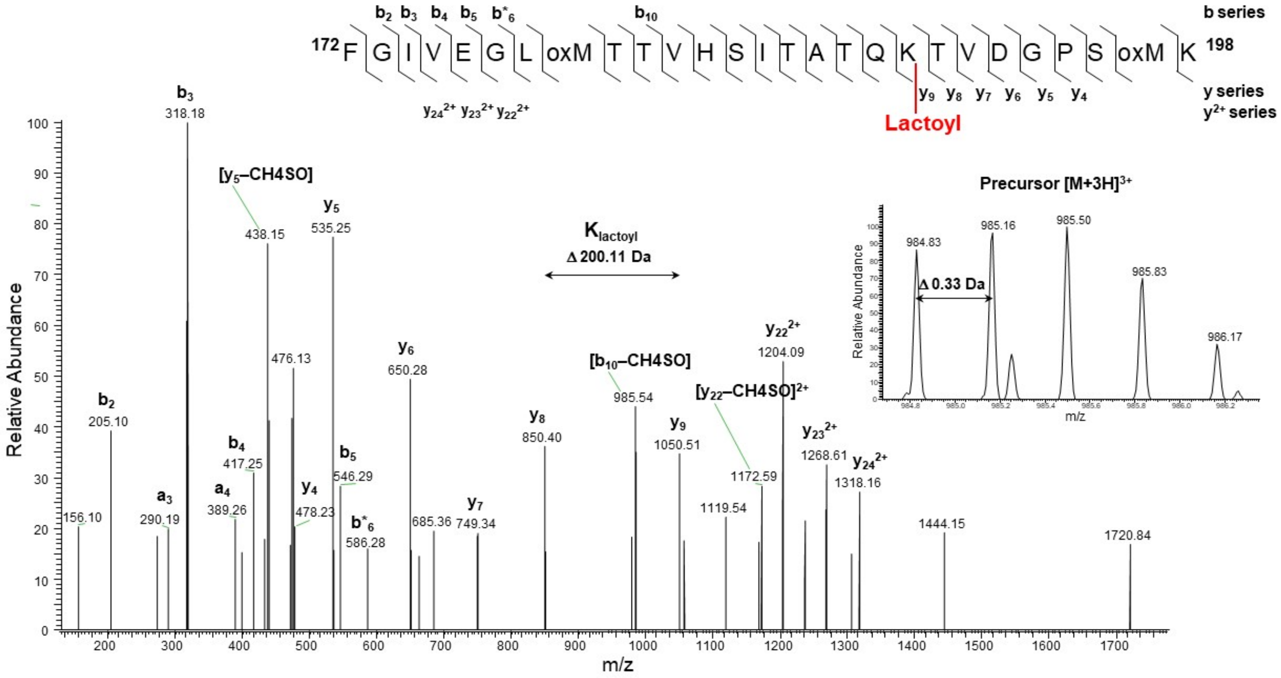

Supplementary Figure S5. NanoLC-MS/MS spectrum of the precursor ion $[M+3H]^{3+}$ at *m/z* 984.83^{3+} fragmented with HCD identifies a *N*-lactoyl Lys modification site at Lys^{190}. Sequence specific b- and y-type fragment ion signals, and a-type ions that derive from the corresponding b-type ion by losing CO, identify the peptide sequence ^{172}FGIVEGLMTTVHSITATQKTVDGPSMK^{198} (top of the panel). Two Met residues denoted oxM were detected in their oxidized forms. The *N*-lactoyl Lys modification site was assigned by the 200.11 Da mass difference between y_8 and y_9 ions of C-terminal peptide fragments. A set of doubly protonated y_{22}^{2+}, y_{23}^{2+}, and y_{24}^{2+} ions provided further evidence for the assignment of lactoyl Lys modification site at Lys^{190} residue. The peak denoted b^* is the result of ammonia (-17 Da) loss from the corresponding ion. Characteristic neutral loss of methanesulfenic acid (CH_3SOH, 64 Da) from the side chain of methionine sulfoxide (oxM) originating from the M^{179} residue was detected for b_{10} ion, and originating from M^{197} was detected for y_5 ion, and for the doubly protonated y_{22}^{2+} ion. The spectral portion of precursor ion MS scan with isotopically resolved peaks indicating the parent ion charge state $[M+3H]^{3+}$ is shown in the insert on the right of the panel.

Supplementary Figure S6

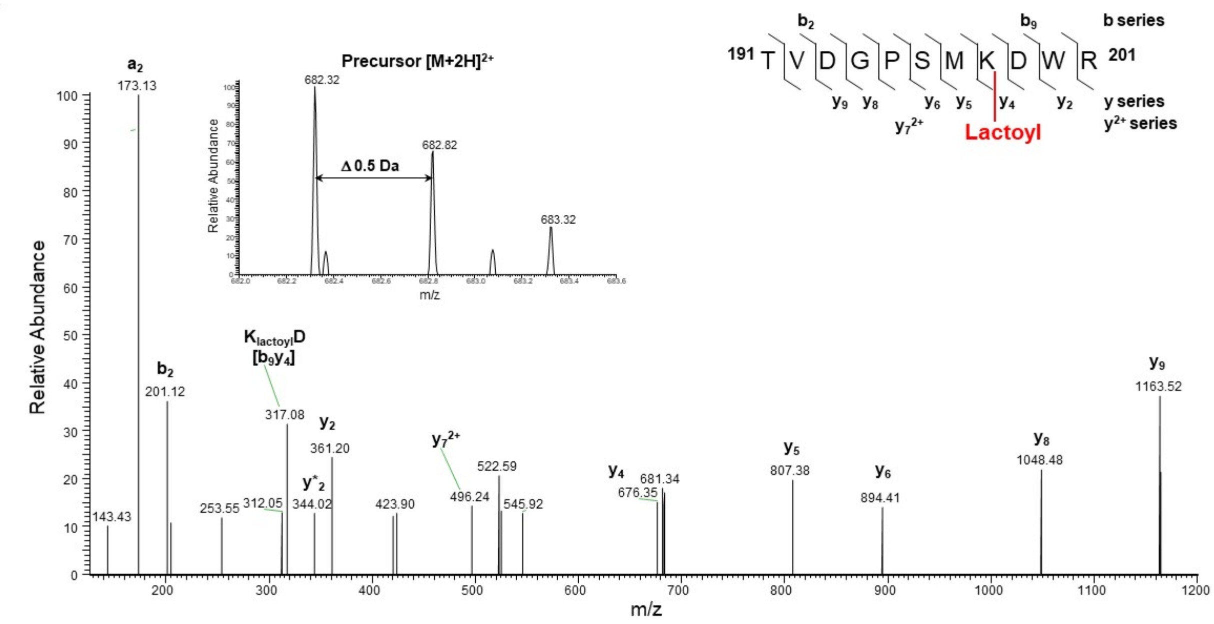

Supplementary Figure S6. NanoLC-MS/MS spectrum of the precursor ion $[M+2H]^{2+}$ at *m/z* 682.32^{2+} fragmented with HCD identifies a *N*-lactoyl Lys modification site at Lys^{198}. Sequence specific b- and y-type backbone fragment ion signals, and a-type ions that derive from the corresponding b-type ion by losing CO, identify the peptide sequence ^{191}TVDGPSMKDWR^{201} (top right corner). The peak denoted y^* is the result of ammonia (-17 Da) loss from the corresponding ion. The internal fragment ion b_9y_4, as well as ions y_4, y_5, y_6, y_7, y_8, y_9, and y_7^{2+} resulting from the peptide backbone fragmentation, provided high confidence evidence for the assignment of lactoyl Lys modification site at Lys^{198} residue. The spectral portion of precursor ion MS scan with isotopically resolved peaks indicating the parent ion charge state $[M+2H]^{2+}$ is shown in the insert (top left corner).

Supplementary Figure S7

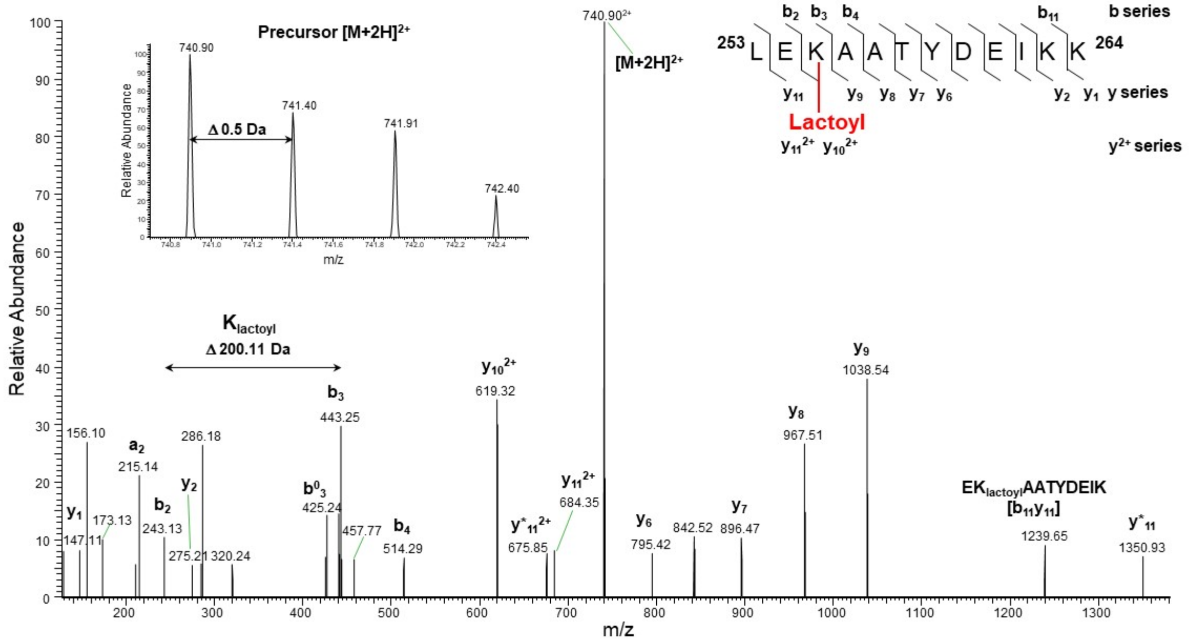

Supplementary Figure S7. NanoLC-MS/MS spectrum of the precursor ion $[M+2H]^{2+}$ at *m/z* 740.90^{2+} fragmented with HCD identifies a *N*-lactoyl Lys modification site at Lys^{255}. Sequence specific b- and y-type backbone fragment ion signals, and a-type ions that derive from the corresponding b-type ion by losing CO, identify the peptide sequence ^{253}LEKAATYDEIKK^{264} (top right corner). The lactoyl Lys modification site was assigned by the 200.11 Da mass difference between b_2 and b_3 ions of N-terminal peptide fragments. The peaks denoted b^0 or y^* are the result of water (-18 Da) or ammonia (-17 Da) loss from corresponding ions, respectively. The internal fragment ion $b_{11}y_{11}$, as well as the sequence specific b_3, b_4, and doubly protonated y_{10}^{2+}, y_{11}^{2+} ions provided high confidence evidence for the assignment of the lactoyl Lys modification site at Lys^{255} residue. The spectral portion of precursor ion MS scan with isotopically resolved peaks indicating the parent ion charge state $[M+2H]^{2+}$ is shown in the top left insert.

Supplementary Figure S8

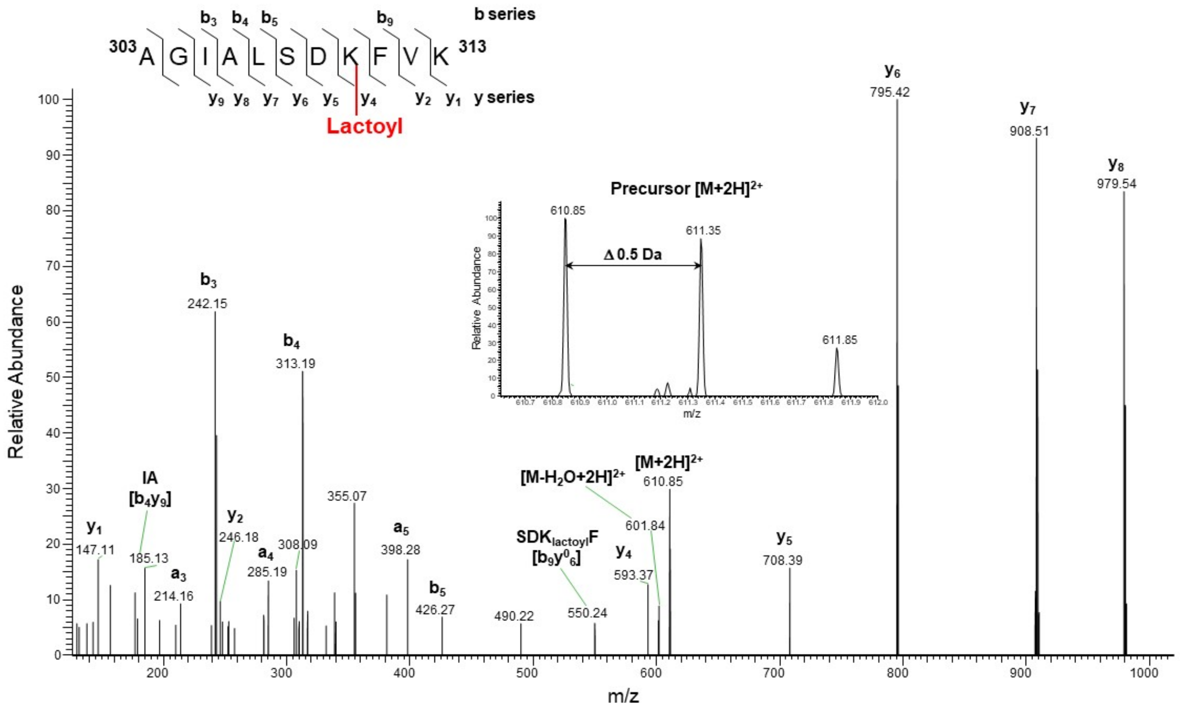

Supplementary Figure S8. NanoLC-MS/MS spectrum of the precursor ion $[M+2H]^{2+}$ at *m/z* 610.85^{2+} fragmented with HCD allowed for identifies a *N*-lactoyl Lys modification site at Lys^{310}. Sequence specific b- and y-type backbone fragment ion signals, and a-type ions that derive from the corresponding b-type ion by losing CO, identify the peptide sequence ^{303}AGIALSDKFVK^{313} (top left corner). The peak denoted y^0 is the result of water (-18 Da) loss from the corresponding ion. The internal fragment ion $b_9y^0_6$, as well as the sequence specific y_4, y_5, y_6, y_7, y_8, and y_9 ions provided high confidence evidence for the assignment of lactoyl Lys modification site at Lys^{310} residue. The spectral portion of precursor ion MS scan with isotopically resolved peaks indicating the parent ion charge state $[M+2H]^{2+}$ is shown in the insert at the center of the panel.

Supplementary Figure S9

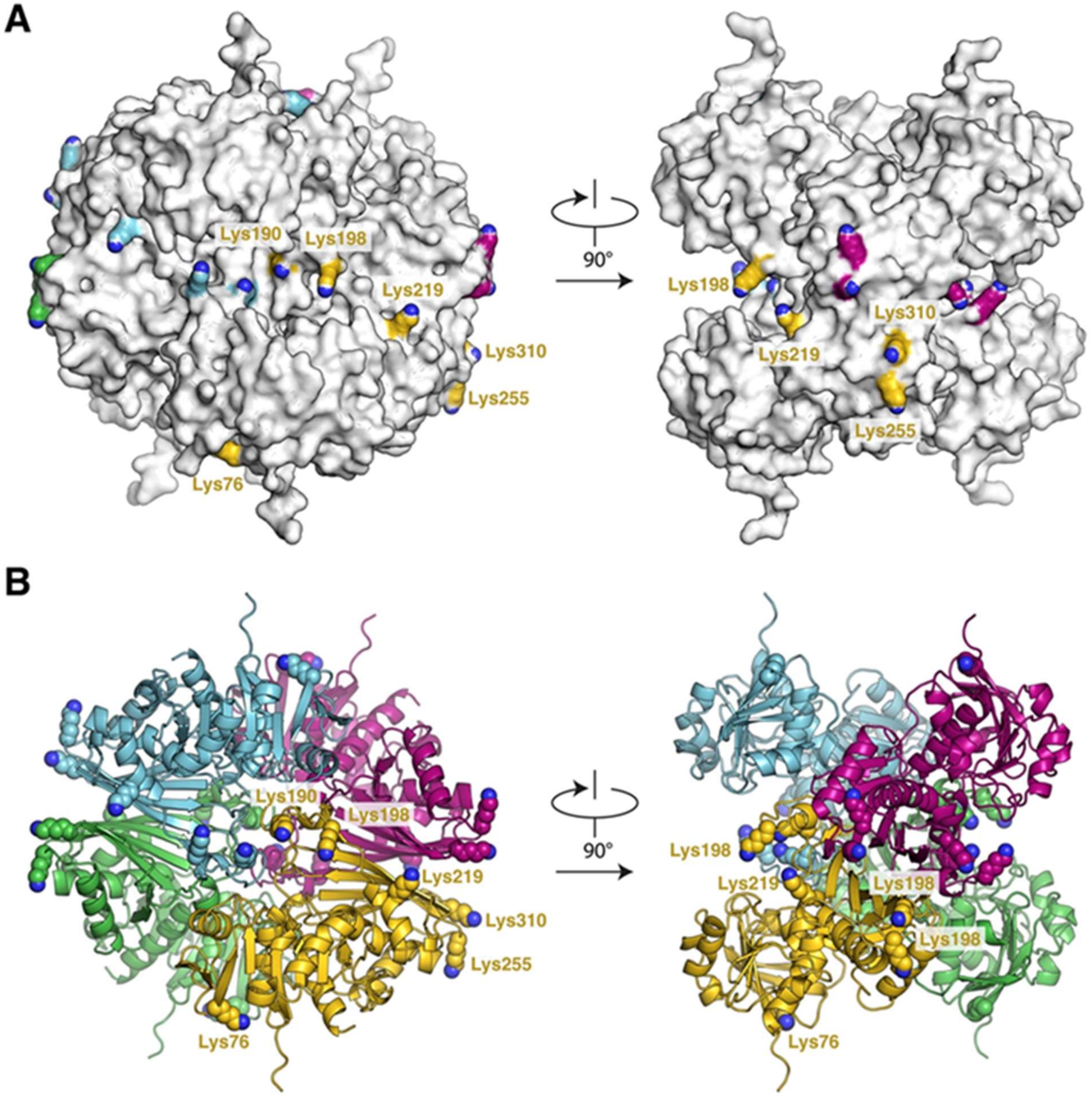

Supplementary Figure S9. Overall tetrameric structure of GAPC1 predicted using AlphaFold3 in (A) surface or (B) cartoon representation. Lysine residues at positions 76, 190, 198, 219, 255 and 310 are in sphere representation with each subunit in a different color.

Supplementary Figure S10

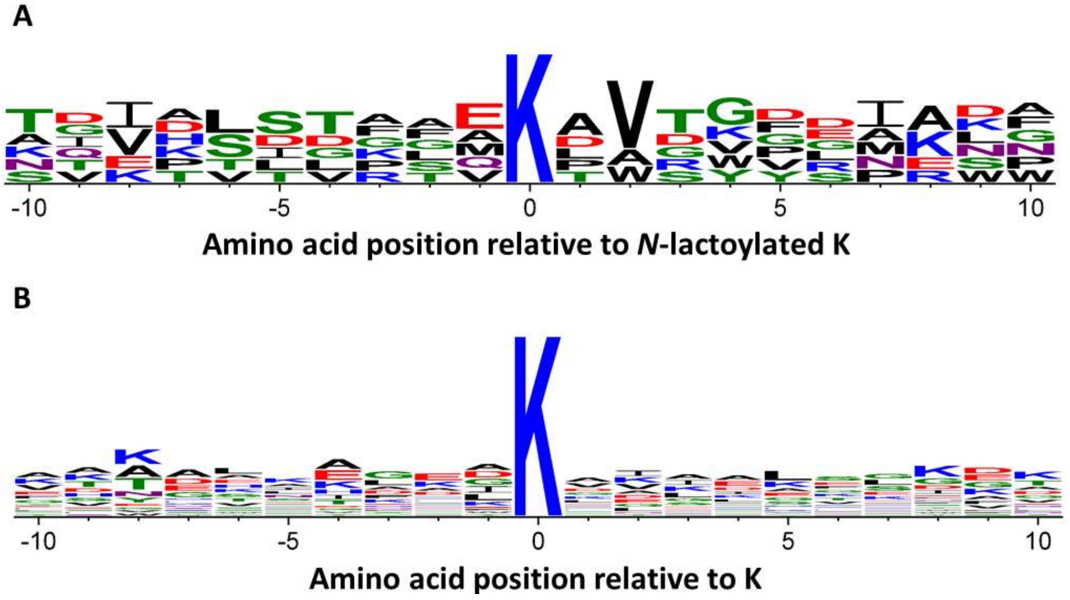

Supplementary Figure S10: Weblogo representation of the amino acid sequence around Lys residues in GAPC1. A: *N*-lactoylated Lys detected in GAPC1. B: Residues that were not detected as *N*-lactoylated. In this case, Lys $^{4,\ 5,\ 63,\ 65,\ 68,\ 117,\ 121,\ 223,\ 255,\ 337}$ were not included in the analysis since they lacked a necessary ten amino acids sequence stretch on both sides or they were not covered in the LC-MS/MS sequencing. The sequence is centered on Lys residues and spans from positions -10 to +10 relative to this residue. The Figure was generated using the Weblogo tool available at https://weblogo.berkeley.edu/logo.cgi.

Supplementary Figure S11

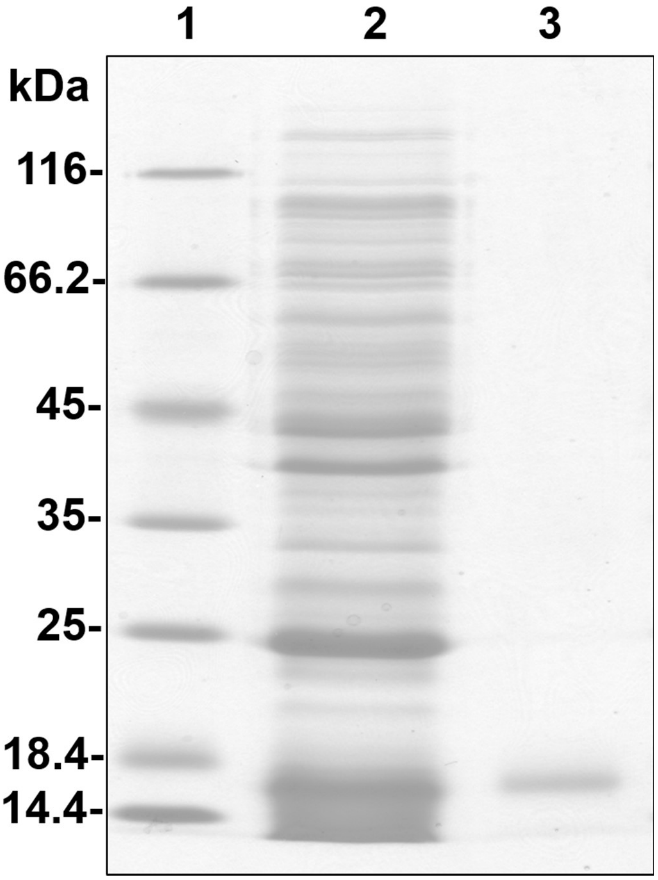

Supplementary Figure S11. SDS-PAGE analysis of the purification of *Arabidopsis thaliana* (6xHis)GRXC1. Lane 1, molecular weight markers; lane 2, protein extract of the IPTG-induced *E. coli* culture; lane 3, purified recombinant (6xHis)GRXC1. Numbers on the left side indicate the running position and size in kDa of molecular weight markers.

Supplementary Figure S12

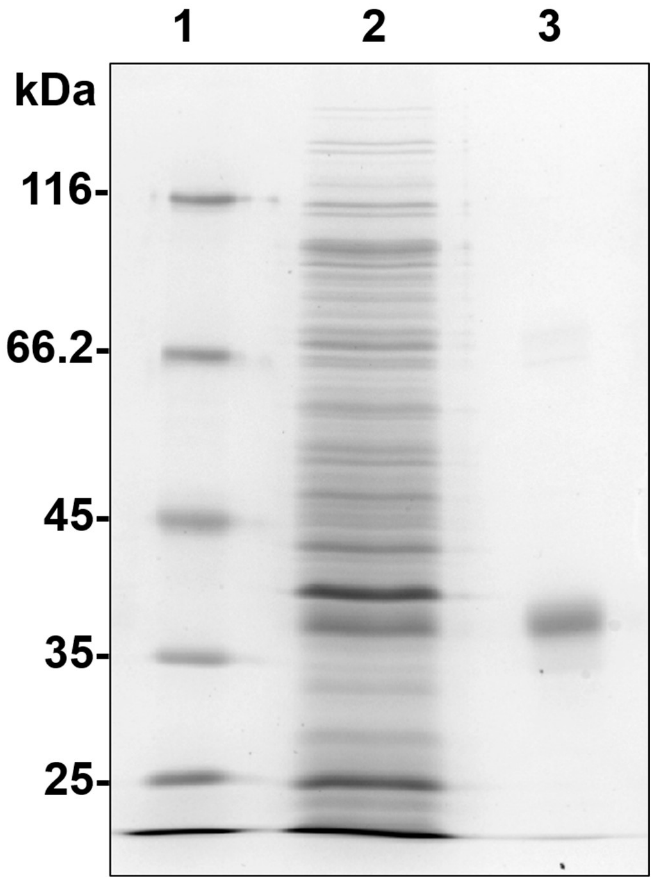

Supplementary Figure S12. SDS-PAGE analysis of the purification of *Arabidopsis thaliana* (10xHis)NTRA. Lane 1, molecular weight markers; lane 2, protein extract of the IPTG-induced *E. coli* culture; lane 3, purified recombinant (10xHis)NTRA. Numbers on the left side indicate the running position and size in kDa of molecular weight markers.

Supplementary Figure S13

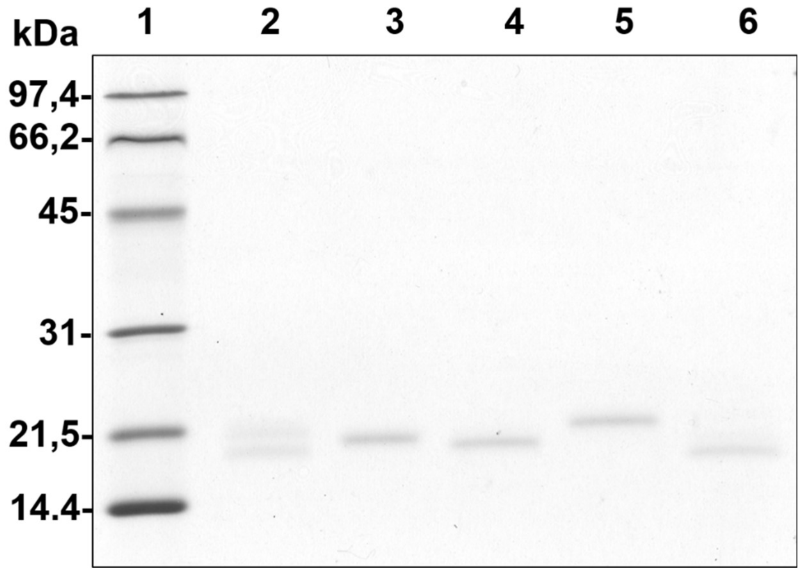

Supplementary Figure S13. SDS-PAGE analysis of purified *Arabidopsis thaliana* recombinant TRXs. Lane 1, molecular weight markers; lane 2, purified recombinant (10xHis)TRX*h*1; lane 3, purified recombinant (6xHis)TRX*h*2; lane 4, purified recombinant (6xHis)TRX*h*3; lane 5, purified recombinant (6xHis)TRX*h*4; lane 6, purified recombinant (6xHis)TRX*h*5. Numbers on the left side indicate the running position and size in kDa of molecular weight markers.
